# Projecting the impact of strengthened tobacco control policy on disparities in US states with persistently high smoking rates

**DOI:** 10.18332/tid/207750

**Published:** 2025-09-18

**Authors:** Emily M. Donovan, Stephanie N. Yoon, Blaine Hardy, Jennifer Kreslake, Michael V. Maciosek

**Affiliations:** 1 Schroeder Institute, Truth Initiative, Washington DC, United States; 2 HealthPartners Institute, Bloomington MN, United States

**Keywords:** public policy, simulation modeling, racial disparities, tobacco control, socioeconomic disparities

## Abstract

**INTRODUCTION:**

Thirteen Southern and Midwestern states – termed ‘Tobacco Nation’ – have persistently higher smoking rates than other US states. Previous research indicates increased cigarette taxes and tobacco control expenditures (TCE) may mitigate this geographical cigarette smoking disparity. The current study simulates the impact of these policies on racial and socioeconomic tobacco-related disparities within Tobacco Nation.

**METHODS:**

Using ModelHealth^TM^:Tobacco, we simulated 20-year changes in smoking and smoking-attributable (SA) outcomes by poverty status and race. We projected the impact of: 1) a ‘tax-only scenario’, increasing cigarette taxes by $1.50; and 2) a ‘combined policy scenario’, simultaneously increasing cigarette taxes by $1.50 and increasing state TCE to the Centers for Disease Control (CDC)-recommended level.

**RESULTS:**

Under the tax-only scenario, SA outcomes would be reduced for Tobacco Nation residents below 138% the federal poverty level (FPL) by about 4.3 the magnitude of those above 138% the FPL. Some SA outcomes would be reduced by about 10% more among Non-Hispanic (NH) Black residents than NH White residents. For all subgroups, the combined policy scenario would reduce SA outcomes by about eight times the magnitude of the tax-only scenario, even though the relative reduction in disparities by poverty status would be smaller (2.8 higher reductions for those below compared to above 138% the FPL).

**CONCLUSIONS:**

The combined policy scenario, compared to the tax-only scenario, would reduce SA harms by a substantially larger magnitude. Both scenarios are projected to reduce socioeconomic disparities in tobacco harms but not all racial disparities in Tobacco Nation without greater prioritization of targeted policies.

## INTRODUCTION

Cigarette smoking prevalence has decreased substantially in the United States (US); from 2005 to 2021, current smoking declined from 23% to 1.9% among high school students and from 20.9% to 11.5% among adults^1-3^. Despite decreases in cigarette smoking over the years, some US residents experience disparate rates of tobacco-attributable disease – particularly low-income individuals, Black individuals, and those living in the Midwest or South^4^. Several environmental factors contribute to tobacco-related disparities. For example, research on sociodemographic disparities demonstrates that there is more tobacco marketing in neighborhoods with lower average incomes and with more Black residents^4,5^. In addition, tobacco control policy data indicate that policies like tobacco taxes, expenditures on tobacco control programs, and smoke-free air laws are weaker in Midwestern and Southern US states^6,7^ .

In 2019, Truth Initiative identified 13 states in the South and Midwest of the US with persistently higher rates of cigarette smoking compared with other US states^8^. Residents of this region, known as ‘Tobacco Nation’, are also more likely to report incomes below the federal poverty level (FPL), more likely to identify as non-Hispanic Black, and less likely to be exposed to tobacco control policies than residents of other US states^8^. Increased enactment of effective tobacco control policies may mitigate geographical cigarette smoking disparities that characterize Tobacco Nation. Policies like cigarette tax increases and increases in tobacco control expenditures (TCE) (i.e. state expenditures for tobacco control efforts such as cessation programs and prevention campaigns) have been shown to reduce tobacco use^9,10^. Our recent simulation study indicates that strengthening such policies in Tobacco Nation could eliminate tobacco-related disparities between the region and other US states^11^.

In addition to reducing geographical disparities between Tobacco Nation and the rest of the US, increases in cigarette taxes and TCE may impact sociodemographic disparities within Tobacco Nation. For example, research demonstrates that individuals with low incomes have been shown to be relatively more responsive to cigarette tax increases compared to those with higher incomes^12^. Additionally, those with lower incomes have been shown to be relatively more responsive than those with higher incomes to population level cessation support^13^. Although there is limited conclusive literature examining tobacco control policy impacts on racial/ethnic disparities, the US Centers for Disease Control (CDC) recommends using TCE to fund tailored cessation programs and campaigns as a strategy to reduce racial/ethnic disparities^9^.

While there have been studies that demonstrated that increased taxes and TCE in Tobacco Nation would reduce geographical smoking-related disparities between Tobacco Nation and other US states^11^, little is known about how these policies would impact smoking-related disparities by race and poverty status. This study simulates the impact of two policy scenarios: 1) Increasing cigarette taxes by $1.50 per pack; and 2) Simultaneously increasing cigarette taxes by $1.50 per pack plus increasing state-level TCE to the CDC-recommended level. Findings from this study can inform policy strategies to reduce disparities in Tobacco Nation.

## METHODS

### Simulation model

We employed a microsimulation model, ModelHealth^TM^: Tobacco, developed in Java. Previous iterations of the model were developed for the US as a whole^14^ and Minnesota^15,16^. The adaptation of the model to Tobacco Nation states has been previously described^17^ and is detailed in Supplementary file Section 1. Here, we briefly describe aspects of the microsimulation model pertinent to the current analysis.

The model simulates annual changes in smoking status and the health and economic harms of smoking by poverty status and race. For the current analysis, we simulated two different age-adjusted populations to facilitate comparisons among population groups within states: one in which the two poverty status groups within each state have the same age distribution as that of the state as-a-whole, and a second in which the race-ethnicity groups have the same age-distribution of the state^18^. We measured household poverty status as a dichotomous indicator of living at or below the 138% of the FPL based on household size and total household income. Race and ethnicity in the simulation model are represented by four broad categories (Hispanic of any race, non-Hispanic Black, non-Hispanic White, and all other races) that are available in the demographic, tobacco use, and disease burden data sets used to parameterize the model.

Within the simulation model, individuals’ poverty status does not change over time. However, keeping poverty status for individuals static as they age does not meaningfully limit population-level estimates of policy effects because the portion of the population living at less than 138% of the federal poverty level is relatively stable across age groups. The age, sex, race and ethnicity, and poverty status composition of the population does change over time in the model due to differences in population composition, as new individuals age into the model and other individuals exit due to death. Individuals who are born into the model as the simulation progresses are assigned the race-ethnicity probabilities of baseline 12-year-olds.

We estimated the probability of youth smoking and net initiation by state of residence, age, sex and race-ethnicity from 2019 Youth Risk Behavior Surveillance System (YRBSS) and we estimated adult cigarette smoking status and cessation probabilities by the same characteristics plus education level and poverty status using combined 2016 to 2019 BRFSS surveys^19,20^. We estimated long-term relapse probabilities from literature as described in Supplementary file Section 1.

The model simulates smoking-attributable (SA) diseases identified in Smoking-Attributable Mortality, Morbidity, and Economic Costs (SAMMEC) as updated in 2014^10^. We obtained incidence and deaths from SA cancers by state, sex, and race/ethnicity from data that inform the US Cancer Statistics Data Visualizations Tool^21^. We obtained deaths for other SA conditions from Detailed Mortality Data^22^. We used hospitalizations to measure annual cardiometabolic and respiratory disease events. We assigned hospitalization rates for each state’s Census Division as tabulated from the 2018 National Inpatient Sample using the Healthcare Cost and Utilization Project (HCUP)^23^. As described in Supplementary file Section 1, we disaggregated cancer incidence and hospitalization rates by smoking status using standard attributable-risk calculations^17^ and the relative risks of mortality of current and former smokers relative to never smokers from the 2014 Surgeon General’s Report^10^.

### Simulation of tax increase scenario

We obtained the average per-pack price of cigarettes by state in 2021 from The Campaign for Tobacco Free Kids (CTFK)^24^. In the tax increase policy scenario, we assume the $1.50 tax adds $1.50 to the point-of-sale price in the first year. We determined the difference in the risk of smoking initiation among youth aged 12–24 years and of smoking cessation among adults over 24 years, in response to a price change based on price elasticity estimates obtained from structured literature reviews (described and referenced in Supplementary file Section 2). Based on the literature review, we used a price elasticity for those at or below 138% of the FPL that is 2.56 greater than the elasticity for those above that threshold. We obtained elasticities for young adults below and above 138% of the poverty level of -0.516 and -0.201, respectively, and -0.295 and -0.115 for adults aged ≥25 years.

We assumed the tax will have a one-time effect on smoking cessation in the year of the increase and an ongoing effect on smoking initiation because new youth and young adult cohorts will be exposed to higher prices as they age into years of risk for starting smoking.

### Simulation of simultaneous tax and tobacco control expenditure increase scenario

A second policy scenario simulates a simultaneous increase in cigarette taxes by $1.50 per pack and an increase in state-level TCE to the CDC-recommended level for each state. Following the literature, the simulation uses state-level TCE as a measure of the intensity of comprehensive tobacco control. We conducted a structured literature review for expenditure elasticities and chose estimates from studies among youth, young adults and other adults which employed the same core statistical analysis for each age group (Supplementary file Section 2)^25-27^. In the simulation, changes from baseline inflation-adjusted per capita TCE results in new smoking initiation and cessation probabilities each simulated year. The literature review did not reveal any consistent modification of the benefit of expenditure increases by socio-economic status or race.

### Conducting the simulation and sensitivity analysis

We compared outcomes in three scenarios: tax increase, simultaneous tax increase and TCE increase, and no policy change (i.e. static policy) scenarios. For every state, we conducted 30 sets of simulations for 1 million individuals, for each scenario with a different random number sequence for each of the 30 sets. To ensure that the difference between the scenarios was attributable only to policy effects, the model used the same random number sequence in each scenario within a set.

We used each state’s 2021 population^28^ to compute weighted-average Tobacco Nation effects for the simulation results of individual states. We then computed a weighted-average for non-Tobacco Nation states as the difference between the US results and the Tobacco Nation average.

Internal validation using model-testing sensitivity analyses ensured that differences in model outputs were consistent with changes to model inputs and with differences in inputs among states and demographic groups. External model validation ensured that the model produced expected population demographics, smoking status, and disease rates consistent with baseline summary statistics from the model input databases and that the outputs of future years were not out-of-bounds of reported effects from similar simulation models. Validation was conducted primarily to ensure that the extensive input tables were correctly populated (providing a second, indirect check on the SAS code that produced the input tables) and that the model’s Java code read the input tables and performed tabulations correctly. No calibration was conducted for model validation or other reasons.

## RESULTS

Baseline adult smoking prevalence estimates, by poverty status and race/ethnicity (NH White, NH Black, and Hispanic), are presented in Table 1 for the US, Tobacco Nation, and Non-Tobacco Nation states. Across the US, cigarette smoking was more prevalent among individuals with lower incomes than those with higher incomes. Cigarette smoking rates by race/ethnicity were less disparate in all regions, with smoking rates generally higher among NH White individuals than NH Black and Hispanic individuals.

**Table 1:** Simulated initial, age-adjusted, adult smoking prevalence by population group in Tobacco Nation, non-Tobacco Nation, and United States, using BRFSS 2016–2019 data^a^

	*Above 138% of* *poverty level* *%*	*Below 138% of* *poverty level ^b^* *%*	*NH White* *%*	*NH Black ^c^* *%*	*Hispanic ^c^* *%*
Alabama	17.55	30.86	25.65	20.06	20.69
Arkansas	17.75	31.06	25.56	20.04	16.09
Indiana	17.29	30.74	24.89	22.63	15.33
Kentucky	22.42	35.74	29.25	24.01	32.19
Louisiana	18.26	32.21	27.11	21.59	25.82
Michigan	15.93	30.57	23.34	22.50	27.87
Mississippi	18.19	31.24	27.66	19.53	22.65
Missouri	16.94	30.48	23.46	25.12	22.34
Ohio	19.90	32.69	26.59	23.62	29.63
Oklahoma	17.87	30.43	24.58	22.47	17.42
South Carolina	15.60	28.62	23.69	19.52	14.74
Tennessee	17.25	30.74	24.84	20.05	21.70
West Virginia	20.57	37.49	28.97	32.84	24.88
United States	14.95	26.16	22.58	20.26	14.63
Tobacco Nation^d^	17.93	31.45	25.42	22.37	23.31
Non-Tobacco Nation^d^	14.12	24.67	21.78	19.67	12.20

a Initial smoking prevalence is derived from the smoking status during the first year of the simulation model with an age distribution for each population group set equal to the age distribution of each state’s overall population.

b Statistically different from individuals above 138% of the federal poverty level at the 99% confidence level in all geographical areas as assessed using Wald chi-squared tests with source data and source weighting while controlling for age distribution. Weights provided in the source data make the survey sample representative of each state.

c Statistically different from non-Hispanic White individuals at the 99% confidence level for Hispanic individuals in all geographical areas and for non-Hispanic Black individuals in all geographical areas except Kentucky, Michigan, and West Virginia as assessed using Wald chi-squared tests with source data and source weighting while controlling for age distribution. Weights provided in the source data make the survey sample representative of each state.

d The Tobacco Nation average is the average of each state weighted by state population. The Non-Tobacco Nation average is computed from the United States average, the Tobacco Nation average and the total population of Tobacco Nation as proportion of the US population.

Table 2 presents the number of SA health events projected per 1 million Tobacco Nation residents, by poverty status and race/ethnicity, aged >20 years, with no policy change. Tobacco Nation residents with incomes below 138% the FPL were projected to experience more SA health events compared to those with incomes above 138% the FPL. While Table 1 indicates baseline smoking was highest among NH White individuals, Table 2 indicates NH Black individuals in Tobacco Nation were projected to experience a greater number of SA health events over 20 years. Hispanic individuals were projected to have the fewest SA health events.

**Table 2:** Simulated Tobacco Nation average^a^ events from smoking-attributable conditions over 20 years without policy change, per one million adults^b^

	*SA cancers*	*SA CVD and* *diabetes* *hospitalizations*	*SA respiratory* *disease* *hospitalization*	*SA cancer* *deaths*	*SA CVD and* *diabetes* *deaths*	*SA respiratory* *disease deaths*	*SA deaths*
Above 138% Federal poverty level	45580	391621	144344	24124	92088	24601	140813
Below 138% Federal poverty Level	53840	425874	171687	28940	96570	29642	155152
White	48313	377618	148358	25417	90622	27370	143409
Black	45427	469756	152295	26123	115779	19194	161096
Hispanic	29252	273631	86045	12026	55142	10392	77559

a The Tobacco Nation average is the average of each state weighted by state adult population.

b Results reflect the initial simulated model population of 1 million in 2021. The population size changes each year in the model. Population groups are age-adjusted to match the overall age distribution of each state. SA: smoking-attributable.

**Figure 1:**
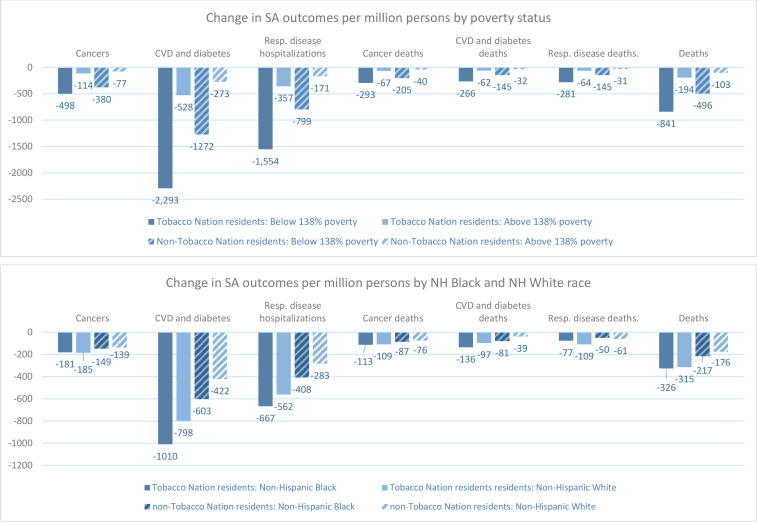
Twenty-year effect of tax increase scenario, compared to static policy scenario, by poverty status and race, per million persons in 2021, age-adjusted^a^; means of simulations for 30 random number seeds

Figure 1 and Table 3 present results for simulations projecting the impact of a $1.50 cigarette pack tax increase compared to a static policy scenario (i.e. cigarette taxes remain at 2021 levels). Results are presented by poverty status (above or below 138% the FPL) and by race (NH White or NH Black) for Tobacco Nation and non-Tobacco Nation residents. Results in Figure 1 indicate that, of the population subgroups examined, Tobacco Nation residents with incomes below 138% the FPL would experience the greatest reductions in SA health outcomes as a result of the tax increase over a 20-year period. Compared to a static policy scenario, estimates indicate there would be 841 fewer SA deaths per million Tobacco Nation residents with incomes below 138% the FPL, while SA deaths for Tobacco Nation residents who have incomes above 138% the FPL, who are NH White, or who are NH Black would decrease by 194, 315, and 326 per million persons, respectively (Figure 1). Ratios in Table 3 indicate that over a 20-year period, the tax increase would reduce the smoking prevalence of Tobacco Nation residents who are below 138% the FPL by 2.5 times the magnitude and SA health outcomes by about 4.3 times the magnitude of Tobacco Nation residents who are above 138% the FPL. Examining results by race, Table 3 indicates that a $1.50 cigarette pack tax increase was projected to reduce smoking rates among both NH White and NH Black individuals in Tobacco Nation and Non-Tobacco Nation states, with NH Black Tobacco Nation residents projected to see smaller reductions in smoking prevalence (0.71 times the magnitude) than NH White residents but 1.2 to 1.4 times greater reductions in some SA health outcomes than NH White residents.

**Table 3:** Relative impacts over 20 years, by poverty status and race, of tax increase scenario, compared to static policy scenario, per million persons in 2021, age-adjusted^a^; means of simulations for 30 random number seeds

*Population*	*Twenty-year* *cumulative effect* *ratio*	*Change in* *smoking* *prevalence* *at* *20 years*	*Change* *in SA* *cancers*	*Change in* *SA CVD and* *diabetes* *hospitalizations*	*Change in SA* *respiratory* *disease* *hospitalizations*	*Change* *in SA* *cancer* *deaths*	*Change* *in SA* *CVD and* *diabetes* *deaths*	*Change* *in SA* *respiratory* *disease* *deaths*	*Change* *in SA* *deaths*
Tobacco Nation residents by poverty status	Below vs above 138% poverty level	2.45	4.37	4.34	4.35	4.37	4.29	4.39	4.34
Non-Tobacco Nation residents by poverty status	Below vs above 138% poverty level	2.49	4.93	4.67	4.68	5.14	4.53	4.71	4.82
Tobacco Nation residents by race	NH White vs NH Black	0.71	0.98	1.27	1.19	1.04	1.41	0.70	1.04
Non-Tobacco Nation residents by race	NH White vs NH Black	0.71	1.07	1.43	1.45	1.14	2.09	0.82	1.24

a Results reflect the initial simulated model population of 1 million in 2021. The population size changes each year in the model. Population groups are age-adjusted to match the overall age distribution of each state. The Tobacco Nation average is the average of each state weighted by state adult population. The Non-Tobacco Nation average is computed from the United States average, the Tobacco Nation average and the total population of Tobacco Nation as proportion of the US population. SA: smoking-attributable. NH: Non-Hispanic.

**Figure 2:**
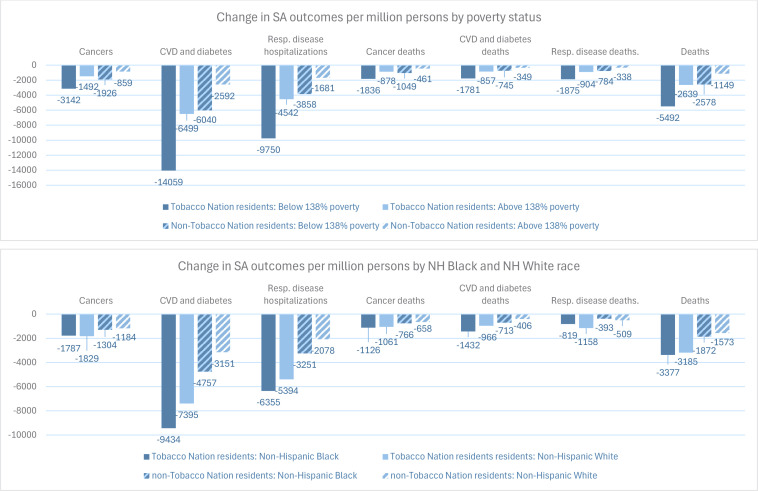
Twenty-year effect of simultaneous tax and tobacco control expenditure increase scenario, compared to static policy scenario, by poverty status and race, per million persons in 2021, age-adjusted^a^; means of simulations for 30 random number seeds

Figure 2 and Table 4 present age-adjusted results for simulation models projecting the impact of simultaneously increasing the cigarette tax by $1.50 per pack and increasing state-level TCE to the CDC-recommended level, compared to a scenario in which cigarette taxes and TCE remain static. Compared to the tax-only scenario, the combined policies would lead to substantially greater reductions in all SA outcomes for all population subgroups examined. Compared to a static policy scenario, the combined policies would reduce SA deaths by 5492 among Tobacco Nation residents with incomes below 138% the FPL, 2639 among residents with incomes above 138% the FPL, 3185 among NH White residents, and 3377 among NH Black residents, per million persons (Figure 2). Similar to the tax-only scenario, Table 4 indicates reductions in SA health outcomes were projected to be greatest among Tobacco Nation residents below 138% the FPL compared with those above 138% the FPL (about 2.1 times greater). Also similar to the tax-only scenario, Table 4 indicates the combined policies would have a greater impact on smoking among NH White individuals than NH Black individuals, but a greater impact on some SA health outcomes for non-Hispanic Black individuals than for non-Hispanic White individuals.

**Table 4:** Relative impacts over 20 years, by poverty status and race, of simultaneous tax and tobacco control expenditure increase scenario, compared to static policy scenario, per million persons in 2021, age-adjusted^a^; means of simulations for 30 random number seeds

*Population*	*Twenty-year* *cumulative* *effect ratio*	*Change in* *smoking* *prevalence at* *20 years*	*Change* *in SA* *cancers*	*Change in* *SA CVD and* *diabetes* *hospitalizations*	*Change in SA* *respiratory* *disease* *hospitalizations*	*Change* *in SA* *cancer* *deaths*	*Change* *in SA* *CVD and* *diabetes* *deaths*	*Change* *in SA* *respiratory* *disease* *deaths*	*Change* *in SA* *deaths*
Tobacco Nation residents by poverty status	Below vs above 138% poverty level	2.76	2.11	2.16	2.15	2.09	2.08	2.07	2.08
Non-Tobacco Nation residents by poverty status	Below vs above 138% poverty level	2.82	2.24	2.33	2.30	2.27	2.13	2.32	2.24
Tobacco Nation residents by race	NH White vs NH Black	0.82	0.98	1.28	1.18	1.06	1.48	0.71	1.06
Non-Tobacco Nation residents by race	NH White vs NH Black	0.86	1.10	1.51	1.56	1.16	1.76	0.77	1.19

a Results reflect the initial simulated model population of 1 million in 2021. The population size changes each year in the model. Population groups are age-adjusted to match the overall age distribution of each state. The Tobacco Nation average is the average of each state weighted by state adult population. The Non-Tobacco Nation average is computed from the United States average, the Tobacco Nation average and the total population of Tobacco Nation as proportion of the US population. SA: smoking-attributable. NH: Non-Hispanic.

### Sensitivity analyses

We previously reported that results for the overall population are most sensitive to different estimates of TCE elasticities, baseline smoking cessation probabilities, the baseline incidence of SA disease, and price elasticities^29^. The sensitivity analysis reported in Table 5 and Supplementary file Tables B–E for Tobacco Nation states indicate, that changes to these variables have little influence on the ratio of effects between population groups when the changes are applied equally to all population groups. In other scenarios, even when the price elasticity is set equal for those above and below the 138% of the poverty threshold, those living below this threshold still benefit more from a tax increase alone (Table 5).

**Table 5:** Sensitivity analysis of equal price elasticities by poverty group for the 20-year cumulative effect of a tax increase alone, compared to static policy scenario, for the Tobacco Nation average ^b^, per million persons in 2021, age-adjusted^a^

*Scenario and population*	*Percent change* *in smoking* *prevalence at* *20 years*	*Change in SA* *cancers*	*Change in* *SA CVD and* *diabetes* *hospitalizations*	*Change in SA* *respiratory* *disease* *hospitalizations*	*Change in SA* *cancer deaths*	*Change in* *SA CVD and* *diabetes deaths*	*Change in SA* *respiratory* *disease deaths*	*Change in SA* *deaths*
**Tax-only scenario, base case with higher price elasticity for those below poverty threshold**								
Below 138% poverty level	-0.87	-498	-2293	-1554	-293	-266	-281	-841
Above 138% poverty level	-0.35	-114	-528	-357	-67	-62	-64	-194
Ratio of 20-year cumulative effect: Below vs above 138% poverty level	2.45	4.37	4.34	4.35	4.37	4.29	4.39	4.34
**Tax-only scenario, sensitivity analysis with price elasticity set equal for those above and below poverty threshold^c^**								
Below 138% poverty level	-0.52	-285	-1278	-882	-166	-154	-160	-479
Above 138% poverty level	-0.41	-145	-663	-471	-84	-85	-84	-253
Ratio of 20-year cumulative effect: Below vs above 138% poverty level	1.26	1.97	1.93	1.87	1.98	1.80	1.90	1.89

a Results reflect the initial simulated model population of 1 million in 2021. The population size changes each year in the model. Population groups are age-adjusted to match the overall age distribution of each state.

b The Tobacco Nation average is the average of each state weighted by state adult population.

c In this scenario, the price elasticity for those above the poverty threshold is increased while that for those below the poverty threshold is decreased such that both are equal to the population-wide elasticity obtained from literature. SA: smoking-attributable. NH: Non-Hispanic.

Our literature review did not indicate that historically disadvantaged individuals were more likely than other individuals to modify smoking behavior with changes in TCE. However, we constructed a scenario to examine what might happen if programs could be targeted such that adults in households below 138% of the federal poverty level were 50% less likely to initiate smoking and 50% more likely to quit if they are smoking, similar to the relative effect of tax increases. The results of this hypothetical scenario (Supplementary file Table C, see 'Expenditure elasticity 50% higher below poverty threshold than above poverty threshold') indicate that simultaneous price and expenditure policies would reduce smoking about 20% more for those below 138% of the poverty threshold compared to the base case (9.5% vs 7.8%).

## DISCUSSION

To our knowledge, this study is the first to use a microsimulation model by US state to project the impact of tobacco control policies on smoking-related disparities by poverty status and race. The current study builds upon our previous work to examine how strengthened tobacco control policies – specifically a $1.50 cigarette pack tax increase alone and a simultaneous increase in cigarette tax ($1.50 per pack) and increase in TCE to the CDC-recommended level – would impact disparities by poverty status and race within Tobacco Nation.

Unsurprisingly, the combined tax and TCE scenario would reduce smoking and smoking harms by a larger magnitude than the tax-alone scenario for all sociodemographic subgroups examined. Both policy scenarios are also predicted to reduce smoking-related disparities by poverty status and reduce some smoking-attributable disparities between NH Black and NH White Tobacco Nation residents. Disparity reductions for NH Black individuals appear to arise from higher baseline risk of SA conditions, not from greater reductions in smoking prevalence. While the tax-only policy scenario is projected to reduce relative disparities by poverty status less than the combined policy scenario, Tobacco Nation residents in all population subgroups examined would experience the greatest reductions in smoking and SA health outcomes from the combined policy scenario.

Few studies have projected how disparities would be impacted by the policies modeled in our study. A calculation in Ukraine suggests that cigarette tax increases would reduce income-based disparities in tobacco-related outcomes^30,31^; however, a simulation study similar to ours in the Netherlands did not find that a cigarette tax increase would affect smoking disparities by education level^32^. In the US, the National Cancer Institute Tobacco Control Monograph 22 reported a comprehensive assessment of the impact of tobacco policy on individuals in the lower two quintiles of US income distribution from the SimSmoke model^33^. That simulation found higher effects of $1.00 and $2.00 tax increases on smoking prevalence over 10 and 30 years compared to no policy change for the first and second income quantiles than we found for those below 138% of the federal poverty level over 20 years. This may be due to a SimSmoke modeling assumption of an immediate effect of a tax increase on smoking prevalence that we did not incorporate. The Monograph did not report the impact of taxes on higher income quintiles. Therefore, we are unable to compare our estimates of disparities reductions to those from that simulation.

### Limitations

The model used in this study is limited by model inputs and assumptions as previously described and evaluated in sensitivity analysis^11^. Notably, our sensitivity analyses did not indicate that changes to model parameters altered the conclusions about relative magnitude of benefits of tax and expenditure policies by population groups. Nevertheless, readers should keep in mind that residual confounding may bias our estimates of some model inputs, including our indirectly tabulated estimates of the burden of tobacco-attributable illness (Supplementary file Section 1) and policy effectiveness derived from literature based, by necessity, on observational studies.

It is important to consider that while we incorporated differences in the risk of smoking-attributable mortality by race and ethnicity, mortality data do not record measures of SES. As a result, any impact of poverty status on smoking-attributable mortality independent of smoking status, age, sex, and race and ethnicity is not reflected in the simulation results. We chose not to incorporate from secondary data any differential mortality from non-smoking-attributable causes of death by poverty status, race or ethnicity. We made this choice to avoid incorporating societal, health system, or environmental bias in a manner that quantifies lower gains in life years and QALYs (quality-adjusted life years) of preventing a smoking-attributable death in disadvantaged individuals^34^. The trade-off is that our results may overstate the actual gains of tobacco control policy for disadvantaged individuals given persistent disparities in life-expectancy.

Additionally, the simulation model was constructed and validated using data from late 2018 to 2021, and therefore we describe our results as covering the 20 years starting in 2021. Smoking rates have continued to fall since 2021 (as predicted by the model), the e-cigarette market and the market for other novel tobacco products continues to evolve, and wage growth for some lower income workers have outpaced inflation in recent years. Therefore, while it is reasonable to expect the absolute benefits predicted for 20 years starting today would be smaller due to lower cigarette smoking prevalence, it is less clear whether the percent reduction in tobacco harms by population groups will be meaningfully impacted by current and future changes to the tobacco use environment.

## CONCLUSIONS

Our study contributes new evidence that, in addition to reducing geographical disparities, increased cigarette taxes and TCE could reduce smoking-attributable disparities by poverty status and some smoking-attributable disparities between NH Black and NH White individuals. Since 2021, the model’s baseline year, tobacco control policies in Tobacco Nation have remained significantly weaker than those in other US states. No Tobacco Nation states increased cigarette taxes during this time, and while Oklahoma increased its TCE to 85.9% of the CDC-recommended level, no other Tobacco Nation state increased its TCE by more than 5% of the CDC-recommended level^35,36^. Policymakers and tobacco control practitioners can use our findings to advance policies that reduce geographical tobacco-related disparities that characterize Tobacco Nation while reducing income-based smoking disparities within Tobacco Nation. To potentially further reduce disparities within Tobacco Nation, policymakers may consider earmarking a portion of state cigarette tax revenue and/or TCE to fund evidence-based tobacco prevention and cessation programs for populations experiencing the greatest burden of tobacco-related disease.

## Supplementary Material



## Data Availability

The data supporting this research cannot be made available for privacy or other reasons. The dataset and code used for this analysis are not available for distribution due to data use agreements between the authors and providers of the source data. We have provided a detailed methodology in the manuscript and in the Supplementary file to facilitate replication of dataset construction and modeling.
