# Supplementary material for: Projecting the impact of strengthened tobacco control policy on disparities in US states with persistently high smoking rates
Source: Tob Induc Dis. 2025 Sep 18;23:10.18332/tid/207750. doi: 10.18332/tid/207750 (PMC12445165; doi:10.18332/tid/207750)
Supplement: Supplementary file 1 [file TID-23-134-s1.pdf]

Supplement 1

HealthPartners Institute

ModelHealth™: Tobacco

Model Health: Tobacco Documentation Model Version 3.1 for a Path Forward for Tobacco Nation

April 29, 2022

Contents

Introduction 3

Model structure 3

Model Initialization and population characteristics 6

The smoking behavior module 7

Initial smoking status *7*

Estimating changes in smoking status *9*

Relapse rates........ 10

Determination of cigarette consumption and tax revenue .... ***Σφάλμα! Δεν έχει οριστεί σελιδοδείκτης.***

The Health Impact Module 12

Disease occurrence and burden estimation *12*

Smoking-attributable mortality risk by age, sex and smoking status 13

Smoking-attributable disease risk by age, sex and smoking status .... **Σφάλμα! Δεν έχει οριστεί σελιδοδείκτης.**

Smoking-attributable diseases, health utilities, and duration........ 14

Use of case fatality rates........**Σφάλμα! Δεν έχει οριστεί σελιδοδείκτης.**

Competing causes of death 16

Costs and productivity........ *16*

Smoking-attributable medical costs 16

Productivity........ 17

Productivity loss from premature mortality........ 18

Productivity loss from absenteeism and presenteeism 18

References 19

Introduction

The HealthPartners Institute ModelHealth™: Tobacco was developed to evaluate the health impact and cost-effectiveness of implementing evidence-based clinical and community preventive services for diverse populations over varying timeframes and from multiple perspectives. Prior versions of ModelHealth: Tobacco has been used to assess clinical, local, state and federal policy changes.[1-7]

ModelHealth: Tobacco estimates the behavioral changes, health and economic impact, and the cost-effectiveness of tobacco control programs and policy. The model employs a flexible microsimulation framework in which the impact of the intervention under analysis is evaluated at the individual level. These individual effects are aggregated to the population level to estimate population health and economic impact.

This version of the model has been updated and reconstructed in Java to decrease model run time, increase versatility, and eliminate compatibility issues with software upgrades. The version allows importation of data for multiple populations. This ability is used in the current report to conduct detailed microsimulation analyses for the US and 13 individual US states.

The version has not yet incorporated some features of prior versions, including health insurance status, smoking intensity, and tabulation of tobacco tax revenues. Those features and others will be incorporated into the next Java version of the model.

This document provides an overview of the base model's structure, the development of the inputs to the base model, and description of the modeling framework and embedded algorithms. Inputs that are specific to clinical interventions, policies, and programs (counseling, tobacco taxes, media campaigns etc.) are discussed in reports specific to their analysis.

Model structure

Overview

ModelHealth: Tobacco is a Markovian individual-based simulation model (i.e. Markov microsimulation). In the healthcare context, a Markov microsimulation simulates individuals aging over time while facing period-specific probabilities ('risks') of changing health behaviors and while experiencing related health outcomes and economic impact. In each cycle (one year in ModelHealth: Tobacco), individuals will either remain in their current state (other than changing age) or transition to a different state. Transition probabilities are obtained from literature and analyses of relevant data. In the model, the state of each individual (age, smoking status, health, etc.) is tracked over time.

The impact of a clinical intervention, program or policy on smoking behavior is determined by comparing the smoking behavior of each agent in a simulation scenario with the policy or program enacted to smoking behaviors in a "baseline" scenario (an environment *without*

policy or program). An individual agent's smoking behavior may or may not change with a change in smoking policies or programs, and if an agent's smoking behavior does change, their health outcomes may or may not be affected. Population-wide effects are determined from summing the experience of all agents, those who do and do not experience change as the result of a policy or program.

For example, *the Community Guide to Preventive Services* recommends increasing the unit price of tobacco products.[8] Some youth who would have started smoking without an increase will not start smoking with a tax increase. Of those who do not start smoking as youth with the tax increase, some will still start smoking as young adults and others will avoid a lifetime of tobacco use. Some smokers never experience significant harms of smoking; some by chance and others by quitting early enough to reduce their risks. For smokers who would never experience harm, avoiding initiation will have no impact on health outcomes. Other would-be smoking initiators will avoid smoking-attributable disease and may have significantly longer lives. Similarly, taxes also increase the probability that current smokers will quit, and whether or not a tax affects a particular smoker's health depends on what would happen to them without a tax increase and how they respond to the tax increase. Through a series of probabilities, the microsimulation produces these heterogeneous individual experiences with and without policy change, and we calculate the population-wide impact by summing these experiences.

Figure S1.1: Structure of HealthPartner's ModelHealth™: Tobacco microsimulation model

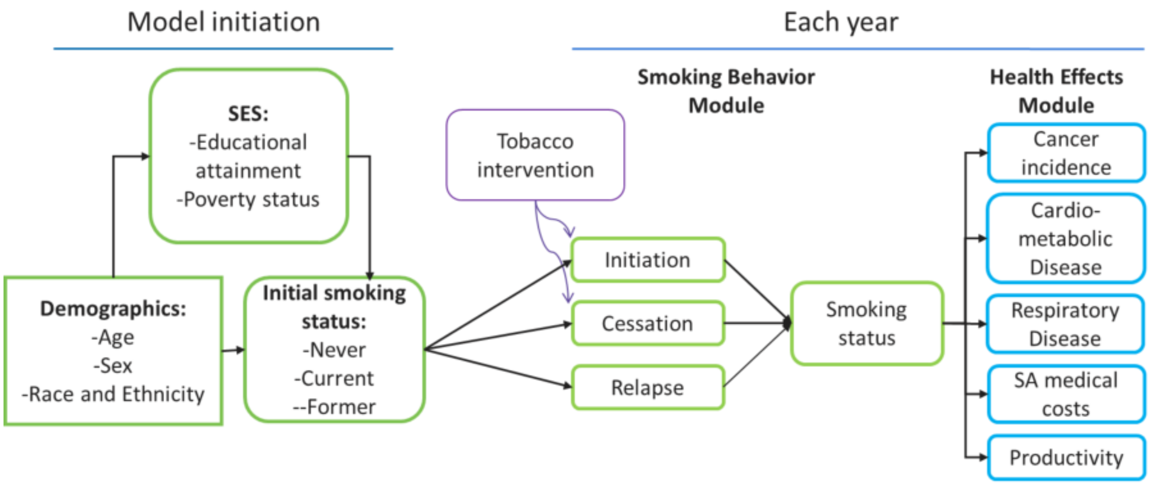

The model can be conceptualized as having three distinct parts, shown in Figure S1.1 and described below. The first part, Model Initiation, defines the population to be modeled and their smoking status. The second part, the Smoking Behavior Module, determines transitions in smoking status over time. The third part, the Health Effects Module, determines health and economic outcomes associated with cigarette smoking.

Software, input-output structure, random number generation and comparison to prior versions

The prior version of ModelHealth: Tobacco was constructed in TreeAge PRO 2015 and employed custom Python and Java code. The current version is built in completely Java, using Microsoft excel to produce population results.

All model inputs have been updated, except for medical costs (they were inflation-adjusted due to data access limitations related to the Covid-19 pandemic). Combined, the input files described below have tens of thousands of entries. Due to their large size, the input tables are not formatted for printing or included in this documentation. They are available on request as comma-separated values (.csv) files. As enumerated below, estimates contained in the files reflect interpolations and extrapolations for population groups needed to inform the model by state. Each source data set has other limitations, noted by the data provider, which are not described below. Therefore, detailed model inputs are available only for use in understanding the simulation model.

This version of the model allows simulations for different populations, such as a state's population or clinical trial participants, by reading a separate set of input files for each population. For this version of the model, input files were created for the US as a whole and each of the 13 Tobacco Nation states identified by the Truth Initiative as having persistently high tobacco use and tobacco harms: Alabama, Arkansas, Indiana, Kentucky, Louisiana, Michigan, Mississippi, Missouri, Ohio, Oklahoma, South Carolina, Tennessee, and West Virginia.[9]

As with prior model versions, the current version can be initialized to simulate a single birth cohort, a dynamic cross-section representative of a particular population, or equally sized strata to be analyzed in weighted analyses to assure adequate size of population subgroups of interest. These different approaches are initialized by using different population input files. In the current analysis, the population input file contains probabilities for each possible age, sex, race-ethnicity, educational attainment, and poverty-status combination (or strata) for the population of interest. For computational speed, Java initially creates a number of individuals for each strata by multiplying the specified simulation population size by the strata's probability. Due to rounding of the probabilities, this initial step leaves a slightly smaller population than specified by the user. Java then fills-in additional simulated agents up to the specified number by randomly selecting a strata to define the individuals' characteristics.

The initial age range for starting population is 12 to 99 years. As the simulation progresses, Java creates new 12 year-olds with the same sex and race-ethnicity probabilities of the initial 12 year-olds (poverty status and educational attainment are assigned later, as described below). To analyze a single cohort, this portion of the code can be disabled or the model output can be filtered to remove the new cohorts. With the code enabled, the model population grows over time. Young cohorts are introduced in numbers that represent their size in current cohorts, which are larger than the older, pre- World War II birth cohorts they replace over time. The model does not incorporate projections of net migration.

To create a model with equally sized strata for weighted analysis, the Java code can be modified to exclude probabilities in the population input table and create equally sized strata. Alternatively, all probabilities in the population input table can be replaced with a constant equal to 1 divided by the number of strata.

Model outputs are generated at three levels: "Micro-level" data of each simulated individual's characteristics, smoking status, and tobacco use consequences for each year simulated; "strata-level" data in which tobacco use and its consequences are aggregated for simulated individuals with common demographic and socio-economic characteristics; and "lifetime aggregate level" in which select outcomes for all individuals are aggregated over the entire simulation period. In the present analysis, micro-level data were used in internal model validation and strata-level data were used to tabulate final results. SAS 9.4 for the Windows operating system was used to summarize micro-level and strata-level outputs, but any statistical software can be used by importing .csv files created in Java. The analyses of data sets to generate model inputs also was conducted with SAS 9.4 for Windows.

This version of the model follows prior versions in pre-specifying the random number sequence such that the same random number draw is used for each possible transition or event in any scenario. This assures that, when comparing scenarios (such as the baseline scenario with a policy scenario) the only difference in outcomes is due to the scenario, while still maintaining random variation among individuals with identical initial characteristics. We have referred to this method as "creating clones," but it has been more formally described and defined by others as the method of "common random numbers"[10].

Population characteristics

ModelHealth: Tobacco starts by generating a population of heterogeneous simulated individuals, or agents. Individuals are assigned up to five demographic and socio-economic characteristics as described below: a single year of age between 12 and 99; male or female sex; one of four broad racial/ethnic groups (black non-Hispanic, Hispanic, white non-Hispanic, and other including multi-racial); one of three broad levels of lifetime educational achievement (no high school diploma, high school degree with or without additional years of education with less than a bachelor's degree, and bachelor's degree or higher); and poverty status (whether or not at or above 138 percent of the federal poverty status).

The model simulates individuals ages 12 and older representative of the age, sex, race-ethnicity, educational attainment and poverty distributions of each state and the US as a whole. Age, sex, and race-ethnicity distributions were tabulated from 2019 US Census Bureau estimates.[11] State-level estimates are top-coded at age 85. Therefore, we distributed each state's total population count at or above age 85 by single year of age using the age distribution of the US population over the same age range, stratified by sex and race-ethnicity.

The probability of being at or above 138% of the federal poverty level and of being in each education status category was estimated using the 2019 Annual Social and Economic

Supplement (ASEC) of the Current Population Survey (CPS).[12] To create estimates for each year of age, sex and race-ethnicity strata, including those strata for which there are no or few persons in the ASEC sample, we used logistic (for poverty status) and multinomial logistic (for education status) regression. We first estimated the probability of poverty status as a function of age, sex, and race-ethnicity, and then estimated education status as a function of age, sex, race-ethnicity, and poverty status. The estimates only assess associations; the order of estimations does not imply that poverty status causes education. In the regression analyses, different age, age-squared and log-age terms were considered along with a complete set of interaction terms. For simplicity and consistency, the set of interaction terms chosen were common across states and based on Akaike information criterion (AIC) in a single regression with state-fixed effects. The functional form for age was allowed to differ by state.

Because poverty status is not available by smoking status for youth at the state level, poverty status in the model is not assigned until age 18. To capture more complete life-time educational attainment, and to facilitate estimation of tobacco use by age range (see below), education attainment in the model is not assigned until age 35. Finally, because CPS data are top-coded at age 80, the logistic and multinomial logistics included only persons through age 79; persons aged 80 or older are assigned the poverty and education status probabilities of 79 year-olds of the same sex, race-ethnicity and state of residence.

Using CPS-ASEC, we determined the probability of being below 138% of the federal poverty status based on total household income and total household size. This is different from the official poverty status measure, which is based on primary family members in the household. However, using household income and size provides better internal consistency with poverty status determined from BRFSS as used elsewhere in the model (see next section). 2019 ASEC-CPS data reflect self-reported income from the prior year. Therefore, we calculate each household's 100% poverty level using the 2018 poverty status definition: $12,140 plus $4,320 for each additional household member after the first for states in the lower 48.

The smoking behavior module

In ModelHealth: Tobacco adults may be in one of three smoking states: *never smoker*, *current smoker* and *former smoker*. Youth (younger than age 18), may be never or current smokers. Cessation and status as former smokers is not tracked for youth in the model due to the experimental nature of youth smoking and associated limitations of the data that quantify youth smoking. Adult smoking status is defined using the accepted criteria of ever having smoked 100 cigarettes:

- **Never smoker:** Having smoked fewer than 100 cigarettes in their lifetime
- **Current smoker:** Having smoked at least 100 cigarettes in their lifetime, and having smoked in the last week

- **Former smoker:** Having smoked at least 100 cigarettes in their lifetime, and not currently a smoker

Initial smoking status

For most states, youth smoking status (current or not current) by age, sex and race-ethnicity was estimated from logistic regression from 2019 Youth Risk Behavior Surveillance System (YRBSS).[13] We created similar estimates from published summaries of the 2018 Indiana Youth Tobacco Survey[14] and Ohio Youth Risk Behavior Survey/Youth Tobacco Survey[15] because 2019 YBRSS data for those states are not available for public download. For Indiana, we assigned smoking rates by single year of age by mapping reported averages by individual grades six through 12 to years 12 through 18. Then, to approximate rates for each age, sex, and race-ethnicity strata, we scaled these individual age estimates by the ratios of female and male smoking rates to overall smoking rates, and the ratios of race-ethnicity smoking rates to overall smoking rates. We made similar calculations for Ohio, except only grades nine through 12 were reported. For younger ages, we calculated the ratio of smoking rates for each year of age from 12 to 14 to the smoking rate at age 15 from the Tobacco Nation states included in the public download YRBSS data and multiplied those ratios by smoking prevalence estimates of Ohio's 15 year-olds.

Using similar multinomial logistic regression methods, we estimated adult cigarette smoking status (never, current, former) as a function of age, sex, race-ethnicity, poverty status and education status from the 2016 to 2019 Behavioral Risk Factor Surveillance System (BRFSS) surveys[16]. Separate equations were estimated for ages 18 through 34, 35 through 51, and 52 through 79, with age ranges chosen to allow sufficient survey responses for each age group and to account for the non-monotonically increasing and decreasing patterns of current and former smoking by age. Due to top-coding of single year of age at 80 years in BRFSS, estimation was limited to survey participants 52 to 79 years of age. The resulting regression coefficients were used to extrapolate the calculation of smoking-status probabilities to age 99. As with other estimations, the form of age and inclusion of interaction terms were determined by AIC.

After initial estimation of probabilities, two additional calculations were made to improve model performance. First, the probabilities based on 2016 to 2019 were rescaled to better reflect the most currently available 2019 smoking rates. This was done by calculating the ratio of the 2019 smoking rates and combined 2016-2019 probabilities by combined strata (to create stable estimates for the single year of 2019) and multiplying the ratio of the 2019 to combined 2016 probabilities to each of the granular strata defined by single-year-of-age, sex, race-ethnicity, education status, and poverty status. The ratios were calculated for each state of residence by sex and poverty status, allowing poverty status to capture a portion of the variation by race-ethnicity and education status.

Second, a relatively simple smoothing was applied at the age cut-offs from the regression segments by using the regression coefficients from both the applicable age range and adjoining age range to estimate probabilities for ages at the age cut-offs and one year removed from the age cut-off. For example, for 33- and 34-year-olds, and also 35- and 36-

year-olds, probabilities were calculated for each strata using coefficients from both the 18 through 34 regressions and 35 through 52 regressions. The two probabilities were then averaged together while giving greater weight to the probabilities based on coefficients from the simulated individual's own age group.

Finally, for simulated individuals who start the model as former smokers, time since quit was assigned to determine the probability of relapse. As described below, a relapse function determines the probability of relapse during the first nine years following the year of a quit. Using BRFSS survey data, we tabulated the portion of former smokers who quit within the last 10 years (year of quit included) and, due to a lack of any clear time pattern in the data, assigned an equal probability of having quit in each of those years. Because the relapse function does not differ by demographic characteristics, the probability of a former smoking having quit within the last 10 years was tabulated only by 10-year age groups and state of residence.

Changes in smoking status

Individuals who have never smoked can either remain in the never smoker state or begin smoking and transition to the current smoker state. A current smoker can remain in the current smoker state or quit and transition to the former smoker state. A former smoker either relapses into the current smoker state or remains in the former smoker state. Figure S1.2 illustrates this conceptual framework of the natural history of smoking tobacco use.

Figure S1.2: Natural History of Cigarette Use

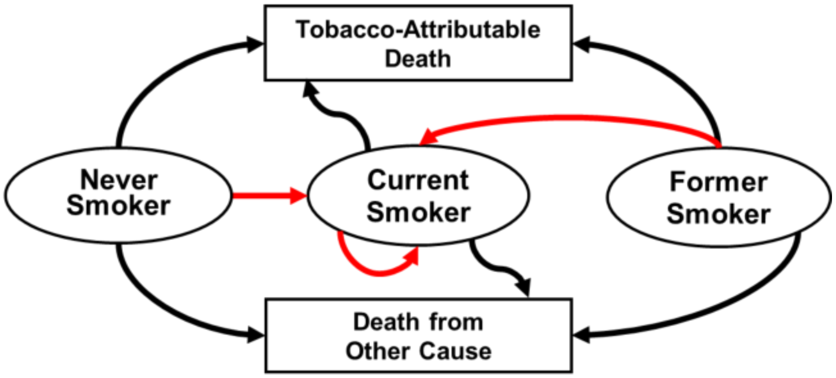

Net initiation

Non-smoking youth ages 12 through 17 have a probability of initiating smoking. That probability is estimated to reflect the probability of initiating smoking, less cessation that does occur during that year of age. This estimate of "net initiation" allows accurate simulation of the prevalence of youth smoking from available data but does not track former smoking status for youth. For each strata, net initiation is simply calculated as the difference between the probability of smoking at the current age, less the probability of smoking at the prior age, and the net initiation probability is calculated as net initiation divided by the probability of being a non-smoker at the prior age. New cohorts of 12-year-olds are introduced to the model with the same probability of smoking as 12-year-olds in

their sex and race-ethnicity strata at model initiation. Because the model does not track smoking behavior before age 12, this probability is, in effect, the probability of having started and continued smoking at any time up to age 12.

Initiation

Young adults, ages 18-24 who are not current or former smokers are assigned a probability of initiating smoking. BRFSS does not ask participants about the age they started smoking or about how long it has been since they started smoking. Therefore, we are not able to directly calculate state-specific estimates of annual initiation rates. Instead, we approximated state initiation rates by first using combined 2015-2018 National Health Interview Surveys (NHIS) to estimate initiation rates by age, sex, and race-ethnicity using logistic regression. We then scaled the resulting national initiation rates by the ratio of the difference in state smoking prevalence between ages 24 and 18 (numerator) and the national differences in smoking prevalence between these ages (denominator). This allows the model to simulate initiation on an annual basis while maintaining consistency with state differences in young adult tobacco use. The ratios were calculated using BRFFSS data and were tabulated and applied by sex and race-ethnicity strata.

Cessation

Each year, current smokers 18 or older are assigned a probability of having quit between 6 and 12 months ago. Cessation probabilities were estimated from BRFSS survey responses. The numerator of the ratio includes survey participants who report being a former smoker at the time of the survey and to have last smoked 6 to 12 months ago. The denominator includes all in the numerator plus current smokers. Cessation probabilities were estimated from logistic regression coefficients as a function of age, sex, race-ethnicity, poverty status, and education level, and with interaction terms chosen according to AIC. Equations were estimated separately for ages 18 to 34 and 35 to 79. Due to top-coding of single year of age at 80 years in BRFSS, estimation of the oldest age group was limited to survey participants 35 to 79 years of age, and the resulting regression coefficients were used to extrapolate the calculation of cessation probabilities to age 99.

Relapse rates

The longer a person has successfully quit smoking, the less likely they are to relapse. We constructed the relapse curve represented by the conditional relapse probabilities shown in Figure 3 based on reporting in five retrospective and prospective studies and reviews.[17-21] We fit a log-linear relapse curve to these estimates as shown in Figure S1.3 for use in the model. These relapse rates are applied to all quits in the model, whether they are part of the baseline model or are induced a clinical intervention, program, or policy change.

In using relapse estimates from the literature, it was important to recognize that the probability of cessation estimated from BRFSS survey responses reflects smokers who quit six to twelve months prior to the survey and remained non-smoking at the time of the survey. Therefore, in applying relapse rates from the literature, we sought an estimate for the first year of relapse that reflected the probability of relapse conditional on having not relapsed during the first six months.

Figure S1.3. Relapse curve – probability of relapse for former smokers

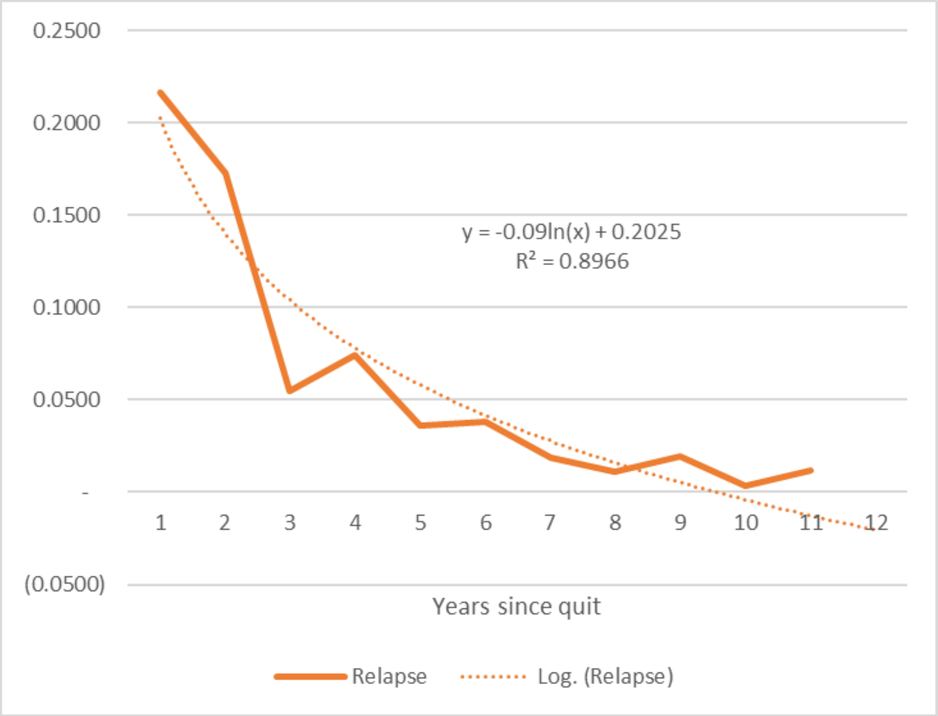

Association between smoking status, poverty status, and education status

When simulated individuals turn age 18 during a model run, they have a smoking status but have not yet been assigned a poverty status. Similarly, simulated individuals who become 35 years old have a smoking status and poverty status but have not yet been assigned an educational attainment level. For internal consistency, the probabilities of poverty and educational attainment are assigned as a function of their current smoking status. BRFSS survey responses were used with logistic regression to estimate the probability that an individual who turns 18 is at or above 138 percent of the federal poverty level as a function of their sex and race-ethnicity and smoking status, estimated separately for each state of residence. To create a more stable estimate with a greater sample size, we used ages 18 to 24 years for the estimation even though the resulting coefficients are used only to assign the poverty status probability to those who turn age 18.

For the purpose of defining the simulated population, the probability of being below 138% of the federal poverty level was determined from CPS-ASEC as described above. This probability was also estimated in BRFSS to create an association between poverty status and smoking. Income in BRFSS is reported in categories. Therefore, we used the method described in Hest 2019 of randomly selecting a specific income from a uniform distribution over the household's reported income category.[22] In doing so, we capped that unbounded highest income category ($75,000 or more) at $235,000 so that the random draw would produce a mean income for this group of $155,000 as used by CDC when determining poverty status from BRFSS responses.[23]

The resulting probabilities of poverty derived from BRFSS were not consistent with the poverty status probabilities derived from CPS-ASEC data due to differences in survey questions. Therefore, we scaled the resulting probabilities by the ratio of poverty status probabilities calculated for the same age group aggregated by sex, race-ethnicity, and state of residence from CPS-ASEC data divided by the same probabilities obtained from BRFSS data.

Similarly, for the purpose of associated tobacco use with educational attainment, using BRFSS data we estimated a multinomial-logistic regressions to obtain the probabilities for the three educational attainment levels used in the model as a function of sex, race-ethnicity, poverty status and smoking status. For these regressions we used survey respondents ages 30 to 44 and used the resulting coefficients to estimate the probabilities of the education attainment levels for those who turn age 35 during a simulation.

Poverty status and educational attainment do not change over time in the current version of the model. Poverty status will change over time in a future iteration of this model, when the health insurance sub-model that was part of previous versions of ModelHealth: Tobacco is updated and ported to Java.

The Health Impact Module

The Health Impact Module determines how the smoking behavior of simulated individuals affects disease incidence, morbidity, and mortality. In assessing policy or program impact, we compare the disease outcomes of each agent that occur in the baseline scenario (without the policy or program) to those that occur in the policy or program scenario. Population-wide estimates of an intervention's impact are determined by aggregating individual effects.

The Health Impact Module tracks outcomes across a variety of tobacco-related diseases using age-, sex-, and smoking-status-based risks derived from the Smoking-Attributable Mortality, Morbidity, and Economic Costs (SAMMEC) as reported in the 2014 Surgeon General's report on tobacco.[24] This approach provides a broad accounting of smoking-attributable risks and diseases. Following SAMMEC, the model includes smoking-attributable events for individuals 35 and older.

Disease occurrence and burden estimation

Cancer incidence

To estimate disease events by smoking status, we first assessed the number and distribution of smoking-attributable disease events by age, sex, and state of residence. Smoking-related incident cancers were obtained from the 2013 to 2017 Surveillance, Epidemiology, and End Results (SEER) Program as prepared the Centers for Disease Control and Prevention and the National Cancer Institute U.S. Cancer Statistics Working Group for the U.S. Cancer Statistics Data Visualizations Tool.[25] Cancers were included if they were identified as having smoking-attributable mortality in the updated SAMMEC presented in the 2014 Surgeon General's report on smoking.[24] Cancers were aggregated into two

categories for which SAMMEC provides relative risks for current and former smokers by age categories: lung cancers and other smoking-attributable cancers (cancers of the lip, pharynx and oral cavity, esophagus, stomach, pancreas, larynx, cervix uteri, kidney and renal pelvis, bladder, and acute myeloid leukemia).

Cancer data by state have some limitations. First, data at the state level are not available by age. Therefore, for each cancer category, we calculated the age distribution of cases by sex and race-ethnicity from US totals and applied those age distributions to sex and race-ethnicity totals for each state. Second, it is not possible to tabulate cases specifically for non-Hispanic whites and non-Hispanic blacks in state data to match the simulation model's race-ethnicity categories. Therefore, rates for whites and blacks with and without Hispanic origin were used. Third, cancer data are not available by poverty status and therefore the simulation model does not differentiate baseline risk of cancers by poverty status. Nevertheless, the simulation results will reflect some differences in disease risk by poverty status to the extent that poverty status is associated with age, sex, and race-ethnicity.

Finally, even with these approximations, some population groups have zero or missing probabilities due to lack of cancer cases or suppressed values in the sample. These include: other cancers for males of other race-ethnicity in Arkansas; lung cancers for both males and females of Hispanic origin and other race-ethnicity in Kentucky; other cancers for both males and females of other race-ethnicity in Mississippi and South Carolina; and both lung and other cancers for both males and females of other race-ethnicity in West Virginia. In these cases, US probabilities for the corresponding cancer and population group were substituted.

Incidence of other smoking-attributable diseases

Events for other smoking attributable conditions for which SAMMEC identifies as having smoking-attributable mortality are measured as hospital admissions with a first-listed discharge diagnosis for the condition in the 2018 National Inpatient Sample (NIS), Healthcare Cost and Utilization Project (HCUP), Agency for Healthcare Research and Quality.[26] This adds seven additional disease categories: ischemic heart disease; other heart disease; cerebrovascular disease; other vascular diseases; diabetes; influenza, pneumonia, and tuberculosis; and chronic obstructive pulmonary disease. The conditions are included in the model for all age groups, but model reports include only the age-group and disease group combinations for which SAMMEC provides relative risks by smoking status. The NIS does not provide state-specific data. Instead, we approximated each state's hospitalization rates with the rates of its Census Division. We tabulated rates by 5-year age group, sex and race-ethnicity.

Cancer mortality

Cancer mortality rates were also obtained from 2013 and 2017 SEER data,[25] and the same limitations and methods described above for cancer incidence are relevant to cancer mortality.

Other smoking-attributable mortality

Deaths for other smoking-attributable conditions (as defined by SAMMEC) were obtained from compressed mortality files, matching on the ICD10 diagnostic codes in SAMMEC.[27] We tabulated mortality rates stratified by 5-year age group, sex, and race-ethnicity. For internal consistency in tabulating case-fatality ratios (see below)rates were tabulated by Census Division rather than individual state. To obtain stable estimates by smoking-attributable condition for the strata, we combined 2010 through 2019 mortality data.

Case-fatality ratios

The simulation used case-fatality ratios for internal consistency so that simulated individuals cannot die from a smoking-attributable condition without having a previous or contemporaneous event from the condition. The ratios are calculated as the number of deaths from a condition in each strata divided by the number of events in the strata. Once a simulated individual experiences a smoking-attributable event, the case-fatality ratio determines the probability that the individual will die from the event.

Event rates by smoking status

Neither SEER cancer data nor the NIS contain cigarette smoking status that could be used to calculate the distribution of disease events by smoking status. We disaggregated cancer incidence and hospitalization rates into never, current and former smokers using standard attributable risk calculations[28-30] and relative risks of mortality from the 2014 Surgeon General's Report.[24] Relative risks are assumed to equal1.0 for ages below 35 in SAMMEC. Therefore, there is no smoking-attributable disease prior to age 35 in the model. The attributable risk calculations are performed inside the Java model to facilitate sensitivity analyses.

The mortality relative risks from SAMMEC were used because relative risks for smoking-attributable conditions for events are not available from a consistent central source. Furthermore, the model assures the relative risks are transferred to smoking-attributable mortality by using the relative risk to calculate event rates for current and former smokers, and then applying case-fatality ratios that are not modified by smoking status. The limitation to this approach is that event relative risks and case-fatality ratios may differ by smoking status, in which case differ event counts by smoking status would be different than what the model produces. However, lacking consistently estimated relative risks and case-fatality ratios, the model's simulation method assures the deaths are apportioned by smoking status according to SAMMEC relative risk. We chose this approach to prioritize accuracy in deaths over total events because deaths are more quantitively important in the calculation of health and economic outcomes.

Smoking-attributable diseases, health utilities and duration of illness

The Health Impact Module independently evaluates the incidence of each disease. Disease-specific quality of life (QofL) decrements are imposed during disease episodes to capture morbidity.

Table 1 lists the diseases included in the health impact module with their assumed duration and quality of life decrement. For model parsimony, disease categories in this update of ModelHealth: Tobacco have been aggregated to those for which relative risks of disease by smoking status are provided in SAMMEC. Therefore, an episode duration and quality of life impairment were chosen to best represent the aggregate category. For internal consistency of health utilities between conditions, we chose utility values from studies that reported utilities for multiple conditions.[31-37]

When a simulated individual has multiple simultaneous health states, health utilities are calculated as multiplicative reductions. This includes living without one of the simulated conditions which was given a health utility of 0.87 [31-34,37]. For example, a condition that is assigned a healthy utility value of .80 relative to an otherwise healthy individual results in a positive utility of 0.872*0.80 = 0.698. Any additional conditions that are present in the same year result in an additional multiplication of each condition's utility value to obtain the total positive utility for the year. The input utilities shown in Table S1.1 reflect the average reduction in utility compared to ideal health (=1.0 – 0.87) so that the average reduction in health utility for otherwise healthy persons relative to ideal health is not double counted during simulation.

For non-fatal cancers, the duration of illness over which a quality-of-life reduction is applied is assumed to average 5 years. For other smoking-attributable conditions, the hospitalization is either treated as an acute event for which the quality-of-life reduction is assumed to be limited to one year (after which another event may occur with another year's quality-of-life reduction), or, for stroke, diabetes and COPD, the hospitalization is assumed to be the start of a chronic condition with lifelong quality of life-reduction.

Table S1.1: Summary of diseases included in ModelHealth: Tobacco

Condition	Episode duration if not terminal	Quality of life decrement
**No smoking-attributable disease**	until death	0.13
**Cancers**		
Trachea, lung, or bronchus	5 years	0.3
Other smoking-attributable cancers	5 years	0.3
**Cardiometabolic disease**		
Ischemic heart disease	1 year	0.23
Other heart disease	1 year	0.23
Stroke	until death	0.25
Other cardiovascular disease	1 year	0.25
Diabetes	until death	0.11
**Respiratory Disease**		
Pneumonia, Influenza, TB	1 year	0.05
COPD	until death	0.2

Time to death

When an event is fatal, the simulation applies a conditional probability of death in year of the event an in each of the subsequent 9 years, such that all fatal events result in death within 10 years. The annual probabilities are conditioned on the event being fatal and the individual having survived to the current cycle. For cancers, these probabilities were determined by age, sex, and race-ethnicity from 10-year survival statistics from SEER 18 areas registry data obtained from SEER Explorer online application.[38] For smoking-attributable respiratory infections, 95.5% of fatalities are assumed to occur in the first year and each subsequent year is assigned a minimal probability of 0.5% to prevent a technical issue of ties when comparing probabilities to a random number draw in the simulation. For other conditions, we assumed the conditional probability of death is a simple inverse exponential function [=1/exp(years since event)], which results in 70% of deaths attributable to the condition occurring in the year of the hospitalization, and 95% of deaths occurring within three years.

A half-cycle correction is applied to the quality-adjusted year of life in the year of death to approximate a mean time of death midway through the year.

Competing causes of death

During each cycle, individuals are also subject to age-specific probabilities of death from other causes. These probabilities are approximated by subtracting the combined probabilities of death from smoking-attributable conditions obtained from compressed mortality data[27] from overall mortality rates by age obtained from U.S. life tables.[39]

Costs and productivity

Model health tracks both direct medical care expenditures and indirect productivity impacts of smoking.

Smoking-attributable medical costs

The model includes only the costs of smoking-attributable medical care. It does not assign medical care costs to never smokers, and the costs of current and former smokers included in the model are net of average-costs for like individuals who are never smokers. For the United States as a whole, we estimated the medical costs of smoking from observed associations between smoking status and medical costs in the Medical Expenditure Panel Survey (MEPS), using smoking status from linked National Health Interview Survey (NHIS) responses.[40] We followed the method of Levy et al.,[41] including controlling for potentially confounding factors in a two-part model using a gamma distribution and a log-link in the second part. However, we combined multiple years of data (2001-2010) to create more stable estimates for age, sex, and smoking status subgroups. It was not possible to create more current estimates due to closures of data-access facilities during the Covid-19 pandemic. For each state, we multiplied estimates of current-smoker SA costs by the ratio of state to U.S. per-capita healthcare expenditures[42] and adjusted these costs to 2021 U.S. dollars using the medical care component of the CPI-U.[43]

MEPS and other claims data are complicated by higher utilization of some former smokers whose quits were prompted by diagnoses that lead to increased healthcare utilization in the years following their successful quits. For former smokers, we fit an exponential function to the relationship of current and former risk based on time since quit, as reported by the Congressional Budget Office (Figure 3-5 in CBO report). We applied this function to the costs for current smokers that we estimated from MEPS data to obtain estimates of what the medical costs of former smokers would be by age, sex and time since quit if they had a proactive quit:

$$y = 0.9927 - 1.086e(-0.1171t) ,$$

where y is the portion of a current smokers' SA costs that is reduced according to years since quit (=t). Thus each former's smoker cost is calculated as a portion of current smokers' costs with the same age, sex and insurance status as estimated from MEPS. The function implies that 50% of the excess medical costs of smoking are eliminated in the 7th year after quit (the functions' "half-life"), and 90% sre eliminated by the 21st year.

The CBO 'index' was constructed based on a literature review of the relationship between time since quit and reduction in mortality risk for smoking-attributable diseases, weighted by each disease's share of smoking-attributable mortality. In their analysis, the CBO applied its index to both mortality risk and medical care expenditures due to lack of better information on the expenditure trend of healthy quitters. Therefore, smoking-attributable medical costs of former smokers based on the function above must be recognized as an approximation. In the current version of ModelHealth: Tobacco, we simplified implementation by assuming applying a time to quit of 5 years for the purpose of estimating former smoker costs for all former smokers (males and females, all ages, all states) which is likely to produce a conservative estimate of cost-savings from quitting.

Productivity

New literature resulted in a substantial change to estimates of productivity losses attributable to smoking in this version of ModelHealth: Tobacco. The simulation model incorporates three sources of productivity loss: premature mortality; absenteeism – days of lost productivity not associated with exit from labor force; and presenteeism – being at less-than-full working capacity during days of work. Each of these categories can have two dimensions: lost labor force productivity and lost non-labor force productivity. Non-labor force productivity could be further divided into time spent producing goods and services outside the formal labor market, and time spent in leisure activity. However, productivity losses in the model are limited to lost labor force productivity and time spent producing services outside of the labor force.

Productivity losses in the model do not yet incorporate reduced labor force participation due to smoking (except that attributable to early morality). A recent article on this topic was published after the simulations for this paper were completed and will be considered in the next model update.[44]

Probability of being employed

The probability of employment by state and age group was obtained from the US Bureau of Labor Statistics.[45] Absenteeism and presenteeism losses are only computed for employed smokers and former smokers.

Productivity loss from premature mortality

The model assigns a positive productivity for each year of adult life, varying by age but not by sex or race-ethnicity. Current and former smokers are assigned a decrement to this productivity to account for absenteeism and presenteeism as described below.

Simulated individuals may experience premature death from smoking-attributable disease. The difference between age of death with and without intervention determines the number of years of premature mortality. We valued the productivity of each year of life using estimates by age group (not differentiated by sex) reported by Grosse et al.[46] updated through 2020 for changes in national average of employee earnings and benefits.[47]

Based on the estimates of Grosse et al.,[46] the model tracks household productivity and workplace productivity separately. Household productivity is assigned to each person in the simulation. Workplace productivity is assigned only to those who are employed even though the estimates provided by Gross et al. are expressed as a per capita rather than a per worker basis. Therefore, the simulation produces a somewhat conservative estimate of productivity losses from smoking. We scaled the nationally representative estimates of Grosse et al. by the ratio of each state's average earnings for all occupation to the US average earnings for all occupations.[48]

Productivity loss from absenteeism and presenteeism

We identified three published studies that assess differences in both workplace and household productivity by smoking status. Those studies allowed us to calculate losses from absenteeism and presenteeism for current and former smokers relative to never smokers, standardized to common number of work hours per year and including the employer costs of benefits and payroll taxes. Using these studies, we calculated mean combined absenteeism and productivity losses of $2,499 (range: $1,343 to $3,970) for current smokers and $669 (range: $379 to $894) for former smokers. We then computed the percent of employment costs lost to smoking-attributable absenteeism and presenteeism by dividing these estimates by average annual employer costs. Finally, the resulting percentages losses were then used to reduce the annual state-adjusted and age specific annual productivity estimates from Gross et al. (-3.02% for current smokers and -0.81% for former smokers).

References

1. Maciosek MV, Flottemesch TJ, Dehmer SP, LaFrance AB, McGree DA, O'Blenes SM. Community Health Advisor. HealthPartners Institute for Education and Research. http://www.communityhealthadvisor.org/drupal/cha/home. Published 2015. Accessed.
2. Maciosek MV, LaFrance AB, Dehmer SP, et al. Health Benefits and Cost-Effectiveness of Brief Clinician Tobacco Counseling for Youth and Adults. *Ann Fam Med.* 2017;15(1):37-47.
3. Maciosek MV, LaFrance AB, Dehmer SP, et al. Updated Priorities Among Effective Clinical Preventive Services. *Annals of Family Medicine.* 2017;15(1):12-22.
4. Maciosek MV, LaFrance AB, St Claire A, Xu Z, Brown M, Schillo BA. Twenty-year health and economic impact of reducing cigarette use: Minnesota 1998-2017. *Tob Control.* 2020;29(5):564-569.
5. Maciosek MV, LaFrance AB, St Claire AW, Keller PA, Xu Z, Schillo BA. The 20-year impact of tobacco price and tobacco control expenditure increases in Minnesota, 1998-2017. *PLoS One.* 2020;15(3):e0230364.
6. Maciosek MV, St Claire AW, Keller PA, LaFrance AB, Xu Z, Schillo B. Projecting the future impact of past accomplishments in tobacco control. *Tob Control.* 2020.
7. Maciosek MV, Armour BS, Babb SD, et al. Budgetary impact from multiple perspectives of sustained antitobacco national media campaigns to reduce the harms of cigarette smoking. *Tob Control.* 2020.
8. The Community Preventive Services Task Force. Interventions to increase the unit price for tobacco products. Reducing tobacco use and secondhand smoke exposure Web site. http://www.thecommunityguide.org/tobacco/increasingunitprice.html Updated November 2012. Accessed.
9. Truth Initiative®. *Tobacco Nation: An Ongoing Crisis.* Washington, DC2019.
10. Stout NK, Goldie SJ. Keeping the noise down: common random numbers for disease simulation modeling. *Health Care Manag Sci.* 2008;11(4):399-406.
11. King M, Ruggles S, Alexander JT, et al. Integrated Public Use Microdata Series, Current Population Survey. https://cps.ipums.org/cps/. Accessed.
12. Bureau of Labor Statistics USCBoLS. Current Population Survey 2019 Annual Social and Economic (ASEC) Supplement. https://www2.census.gov/programs-surveys/cps/datasets/2019/march/asecpub19sas.zip. Accessed Sep 09, 2020.
13. Centers for Disease Control and Prevention. 2019 Youth Risk Behavior Surveillance System (YRBSS). https://www.cdc.gov/healthyyouth/data/yrbs/index.htm. Published 2019. Accessed Sep 30, 2020.
14. Tobacco Prevention and Cessation Commission, Indiana State Department of Health. Results from the 2018 Indiana Youth Tobacco Survey. 2019.
15. Ohio Department of Health. 2019 Youth Risk Behavior Survey Results. Ohio High School Survey. Summary Tables - Weighted Data.
16. Centers for Disease Control and Prevention. Behavioral Risk Factor Surveillance System. Annual Survey Data. https://www.cdc.gov/brfss/annual_data/annual_data.htm. Accessed May 02, 2021.
17. U.S. Department of Health and Human Services. *The Health Benefits of Smoking Cessation.* Vol DHHS Publication No. (CDC) 90-8416. Rockville, MD: U.S. Department of

Health and Human Services Public Health Service Centers for Disease Control Center for Chronic Disease Prevention and Health Promotion Office on Smoking and Health; 1990.
18. Wetter DW, Cofta-Gunn L, Fouladi RT, Cinciripini PM, Sui D, Gritz ER. Late relapse/sustained abstinence among former smokers: a longitudinal study. *Prev Med.* 2004;39(6):1156-1163.
19. Herd N, Borland R, Hyland A. Predictors of smoking relapse by duration of abstinence: findings from the International Tobacco Control (ITC) Four Country Survey. *Addiction.* 2009;104(12):2088-2099.
20. Hughes JR, Peters EN, Naud S. Relapse to smoking after 1 year of abstinence: a meta-analysis. *Addict Behav.* 2008;33(12):1516-1520.
21. Gilpin EA, Pierce JP, Farkas AJ. Duration of smoking abstinence and success in quitting. *J Natl Cancer Inst.* 1997;89(8):572-576.
22. Hest R. Four Methods for Calculating Income as a Percent of the Federal Poverty Guideline (FPG) in the Behavioral Risk Factor Surveillance System (BRFSS). 2019, State Health Access Data Assistance Center, University of Minnesota.
23. Centers for Disease Control and Prevention. *Statistical Brief on the Health Care Access Module, 2013 and 2014.* 2015.
24. U.S. Department of Health and Human Services. The Health Consequences of Smoking—50 Years of Progress: A Report of the Surgeon General. In. Atlanta, GA: U.S. Department of Health and Human Services, Centers for Disease Control and Prevention, National Center for Chronic Disease Prevention and Health Promotion, Office on Smoking and Health; 2014.
25. U.S. Cancer Statistics Data Visualizations Tool, based on 2019 submission data (1999–2017).): U.S. Department of Health and Human Services, Centers for Disease Control and Prevention and National Cancer Institute,. https://www.cdc.gov/cancer/uscs/dataviz/download_data.htm. Accessed Aug 02, 2020.
26. National Inpatient Sample (NIS). Healthcare Cost and Utilization Project (HCUP). In: Agency for Healthcare Research and Quality; 2018.
27. Centers for Disease Control and Prevention. Compressed Mortality File 1999-2006. CDC WONDER On-line Database, compiled from Compressed Mortality File 1999-2006 Series 20 No. 2L, 2009. http://wonder.cdc.gov/cmf-icd10.html. Accessed Dec 8, 2009 12:52:14 PM.
28. Maciosek MV, Xu X, Butani AL, Pechacek TF. Smoking-attributable medical expenditures by age, sex, and smoking status estimated using a relative risk approach. *Prev Med.* 2015;77:162-167.
29. Greenland S. Concepts and pitfalls in measuring and interpreting attributable fractions, prevented fractions, and causation probabilities. *Ann Epidemiol.* 2015;25(3):155-161.
30. Eide GE, Heuch I. Average attributable fractions: a coherent theory for apportioning excess risk to individual risk factors and subpopulations. *Biom J.* 2006;48(5):820-837.
31. Nyman JA, Barleen NA, Dowd BE, Russell DW, Coons SJ, Sullivan PW. Quality-of-life weights for the US population: self-reported health status and priority health conditions, by demographic characteristics. *Med Care.* 2007;45(7):618-628.
32. Mittmann N, Trakas K, Risebrough N, Liu BA. Utility scores for chronic conditions in a community-dwelling population. *Pharmacoeconomics.* 1999;15(4):369-376.
33. Fryback DG, Dasbach EJ, Klein R, et al. The Beaver Dam Health Outcomes Study: initial catalog of health-state quality factors. *Med Decis Making.* 1993;13(2):89-102.
34. Sullivan PW, Ghushchyan V. Preference-Based EQ-5D index scores for chronic conditions in the United States. *Med Decis Making.* 2006;26(4):410-420.

35. Salomon JA, Haagsma, J.A., Davis, A., de Noordhout, C.M., Polinder, S., Havelaar, A.H., Cassini, A., Devleesschauwer, B., Kretzschmar, M., Speybroeck, N., Murray, C.J., Vos, T. . Disability Weights for the Global Burden of Disease 2013 study. *Lancet Glob Health* 2015 3(11):e712-723.
36. Sullivan PW, Lawrence WF, Ghushchyan V. A national catalog of preference-based scores for chronic conditions in the United States. *Med Care.* 2005;43(7):736-749.
37. Gold MR, Franks P, McCoy KI, Fryback DG. Toward consistency in cost-utility analyses: using national measures to create condition-specific values. *Med Care.* 1998;36(6):778-792.
38. National Cancer Institute. SEER*Exploer. seer.cancer.gov/explorer/application.html. Published 2021. Accessed Jun 28, 2021.
39. Arias E. *United States Life Tables, 2010.* Centers for Disease Control and Prevention, Division of Vital Statistics; November 6, 2104 2014.
40. Chowdhury S MS, Wun L. *Linking Medical Expenditure Panel Survey to the National Health Interview Survey: Weighting and Estimation. Working Paper No. 12005.* Rockville, MD: Agency for Healthcare Research and Quality;2012.
41. Levy DE, Newhouse JP. Assessing the effects of tobacco policy changes on smoking-related health expenditures. In: Bearman PS NK, Wright L, ed. *After Tobacco: What Would Happen If Americans Stopped Smoking?* New York: Columbia University Press; 2011:256–289.
42. Centers for Medicare & Medicaid Services. Health Expenditures by State of Residence. Centers for Medicare & Medicaid Services. http://www.cms.gov/NationalHealthExpendData/downloads/resident-state-estimates.zip. Published 2011. Accessed 4/27/2017.
43. Consumer Price Index-All Urban Consumers. U.S. Bureau of Labor Statistics; 2022. https://www.bls.gov/cpi/data.htm. Accessed 03/31/2021.
44. Shrestha SS, Ghimire R, Wang X, Trivers KF, Homa DM, Armour BS. Cost of Cigarette SmokingAttributable Productivity Losses, U.S., 2018. *Am J Prev Med.* 2022.
45. States: Employment status of the civilian noninstitutional population by sex, race, Hispanic or Latino ethnicity, marital status, and detailed age, 2019 annual averages. U.S. Bureau of Labor Statistics; 2020. https://www.bls.gov/lau/table14full19.htm. Accessed Aug 08, 2021.
46. Grosse SD, Krueger KV, Pike J. Estimated annual and lifetime labor productivity in the United States, 2016: implications for economic evaluations. *Journal of medical economics.* 2019;22(6):501-508.
47. Bureau of Labor Statistics, US Department of Labor. Employer Costs for Employee Compensation. Total compensation cost per hour worked for civilian workers Web site. https://data.bls.gov/timeseries/CMU1010000000000D. Accessed Aug 08, 2021.
48. Bureau of Labor Statistics, US Department of Labor. Occupational Employment and Wage Statistics (OEWS) Survey. https://www.bls.gov/oes/#data. Accessed Aug 08, 2021.

Supplement 2

HealthPartners Institute

ModelHealth™: Tobacco

Background and Model Implementation of Policies for a Path Forward for Tobacco Nation, Health Equity

April 29, 2023

© 2019 HealthPartners Institute

Contents

Tobacco Taxes 2
Background 2
Approach of similar models 2
ModelHealth:Tobacco approach 3
Price elasticity literature 3
Increasing Investments in Tobacco Control 6
Background 6
Expenditure elasticity literature 6
Calculation of cumulative expenditures 7
Applying elasticities to changes in smoking status in the simulation 10
The relationship between elasticities and relative risk 10
Policy initiation rates approximated using elasticity 11
Policy cessation rates approximated using elasticity 11
Adjustment to avoid double-counting long-term relapse in the simulation 12

Tobacco Taxes

Background

Extensive literature exists on the effect of tobacco taxes. Most of the literature expresses results in terms of price elasticities: the percentage change in a smoking behavior that occurs with each percent change in price. The most common behavior measured is smoking prevalence, though measures of initiation and cessation have been reported. Tax elasticities have also been reported (the percent change in smoking behavior that occurs with each percent change in *tax)*, but most estimates are price elasticities. Studies also have estimated smoking-intensity elasticities - the percentage change in cigarettes smoked among smokers who continue to smoke. Estimates of the effect of taxation based on elasticities typically use price elasticities and assume that 100% of the tax increase is passed on to consumers as an equal increase in price. The literature has explored several topics, many without clear conclusion, including whether elasticities differ by SES and age, the impact of smuggling to avoid taxes, whether elasticities have decreased over time, and the distributional impact of tobacco taxes. The International Agency for Research on Cancer (IARC) published an extensive review of the impact of tobacco taxes in 2011[1], and the Guide to Community Preventive Services updated the IRAC's review of price elasticities in 2014.[2]

Approach of similar models

Price elasticities are typically measured with smoking prevalence as an outcome. Prevalence is determined by the underlying propensities to initiate, quit, and resume smoking. In some models, the effects of a policy change can be approximated through a one-time change in smoking prevalence. However, in a microsimulation model such as HealthPartners Institute's ModelHealth™:Tobacco, prevalence changes occur through changes to cessation or initiation probabilities. We reviewed the approach taken by similar models to identify best practice for estimating tax impacts. We identified three simulation models that implemented tobacco tax impacts through initiation and cessation transitions rather than through direct change in prevalence: SimSmoke as described in Levy et al.;[3] the RIVM chronic disease model (CDM) as described by van Baal et al.;[4] and the Congressional Budget Office's (CBO) analysis of a federal tax increase.[5] The approaches used by these three models to estimate the impact on taxes through price elasticities are summarized in Table S2.1.

Table S2.1. Approach for modifying initial and ongoing effects of a tobacco tax increase in published analyses				
	**Modeled impact of tax increase through smoking behavior? (Yes/no)**			
**Study**	**Initial prevalence[a]**	**Ongoing initiation**	**Ongoing cessation**	**Relapse**
SimSmoke (Levy et al. 2000)	Yes	Yes	Yes	no
CDM (van Baal et al. 2007)	Yes	no	no	no
CBO 2012	Yes	Yes	no	no
[a]Modeled either directly or through an initial impact on adult cessation				

Models have assumed that the tax impact on prevalence among youth and young adults operates through either initiation or "net initiation". The tax impact on older adults operates through cessation. Each of the three models summarized in Table S2.1 includes an initial change in prevalence operating through cessation at the time of the tax increase. Levy et al. also modeled an ongoing effect on cessation. Unlike the other models, Van Baal et al. excluded an ongoing impact on initiation while noting this is a conservative assumption. None of the models assumed an impact of taxes on relapse.

ModelHealth:Tobacco approach

We follow the CBO approach of implementing a one-time change in the cessation rate for adults ages 25 and older, and a permanent change in initiation rate. The change in initiation rate is applied to non-smokers ages 9-25. Modeling a permanent impact of a tax increase on the probability of smoking initiation accounts for the impact of the tax as new birth cohorts age into the years of increasing risk for tobacco use.

Price elasticity literature

We reviewed price elasticity estimates included in the systematic review reported in IARC Handbooks of Cancer Prevention, Volume 14.[1] Among the cited US studies, we considered those based on person-level data because they use more recent data and more rigorous methods. We included one additional US-based person-level study identified in the Community Guide's supplemental search to the IARC review.[2] We calculated average elasticity estimates across included studies. When multiple studies used the same data source (such as the National Health Interview Survey) and with largely overlapping data years, we selected one study to prevent one data source from heavily influencing average elasticity

estimates. Typically, we chose the study with the most data years or the most recent study. A second study was considered if analysis method and results differed substantially from others using that data source.

We separately considered estimates for youth[6-17] and adults.[9,18-23] Among adults, we studied variation in elasticity by age group and found compelling evidence that adults younger than age 25 were more responsive to cigarette price changes than older adults. While some evidence indicates that middle-aged adults respond differently to price changes than older adults, the evidence was limited and not consistent and therefore we did not further stratify elasticities by age group. The resulting estimates of elasticities and their use in the simulation model are summarized in Table S2.2. The reported estimates for ages 25+ are based on studies that reported results by age group[9,18-20], allowing us to derive an estimate that excludes younger adults. We explored an estimate using a different set of studies that included adults of any age.[18,21-23] The resulting estimate for prevalence elasticity (-0.21) was consistent with the expectation of greater price responsiveness when including younger adults. However, a similar estimate we created for smoking intensity elasticity (-0.11) was lower than the estimate based on studies excluding younger adults. In the version of ModelHealth:Tobacco created for this study, we did not estimate the impact of price on smoking intensity.

Table S2.2. Prevalence elasticities with respect to tobacco price used in ModelHealth:Tobacco

Age group	Elasticity Estimate	Use in model
**9-17**	-0.63	Permanent change in initiation
**18-24**	-0.28	Permanent change in initiation
**25+**	-0.16	One-time change in prevalence, operating as a temporary change in cessation rate

We conducted a second literature search was to determine the relative differences in price elasticity for tobacco use by poverty status and race or ethnicity. We did not find consistent evidence that price elasticity varies by race or ethnicity. Therefore, here we describe only the literature we used to estimate the relative difference in price elasticity by poverty status that was incorporated into the simulation model.

We started by examining 45 articles identified in two systematic reviews[24,25] covering the literature from 1995 through 2018. We sought studies that examined the association between tobacco price elasticity and poverty or income with participation (any current use) as the tobacco use outcome. The reviews included no studies on the relationship between poverty status and price elasticity that met that criterion. However, they included seven studies for which we could compute the ratio of price elasticities between lower and higher income groups [26-32] These studies were conducted among US or Canadian populations. We excluded one study from Australia[33] in which the ratio of elasticity of low-income to higher-income individuals was more than twice that of any study from the US or Canada, and we excluded a study from the US in which the ratio could not be computed because no price-effect was found for the upper income group.[18] We identified one additional study by conducting a supplement search for recent articles and articles that may have been missed by the two reviews.[34] The ratio of lower-income to higher-income price elasticities among the eight included studies was 2.563 (range 0.097 to 4.333).

All studies were among adults. The most common income group comparison included was the lowest income quartile compared to the three higher income quartiles (4 of 8 included studies).[30-32,34] One study compared the lowest income tercile to the two higher income terciles.[28] One included study compared between those below vs above the median income (e.g. two lowest quartile compared to two highest quartile).[27] One study compared a the lowest and middle income groups combined to a 3rd higher income group, in which the lowest and middle income group comprise approximately 60% of the observations.[26] The final study compared those with household incomes of less than $35,000 to all those with household incomes above that threshold, which, at the time of the data collection, would mean those with household incomes in the lowest 40% were compared to those with household incomes in the highest 60%.[29]

The range of elasticities ratios for the 4 studies which compared the lowest quartile to the three upper quartiles was 2.000 to 2.838, which is narrower than the overall range and squarely captures the mean ratio of 2.563. The ModelHealth:Tobacco microsimulation model simulates individuals with household income below versus above 138% of the federal poverty. With approximately 25% of the US population having household income below 138% of the federal poverty level, the 138% threshold aligns well with the studies which compared those in the lowest quartile to those in three higher quartiles of income.

Therefore, we used the elasticity ratio of 2.563 to calculate the price elasticities for those living at or below 138 percent of the federal poverty and those living above that threshold given that approximately

25 percent of the population living at or below the threshold. We solved the equation $E_T = E_{BP}*P + E_{AP}*(1-P)$ for E_{BP} and E_{AP}, where E_T is the population-wide (all income levels) elasticity shown in Table S2.2, E_{BP} is the elasticity for those at or below 138% of the federal poverty level, E_{AP} is the elasticity for those above 138% of the federal poverty level, and P is the percent of the population at or below 138% of the federal poverty level, which we set equal to 25%. For young adults, we obtained $E_{BP} = -0.516$ and $E_{AP} = -0.201$. For adults 25 and older we obtain $E_{BP} = -0.295$ and $E_{AP} = -0.115$.

There are no state data on tobacco use by poverty status for youth. Therefore, in the simulation, tax effects are assumed to be the same for youth below and above 138 percent of the poverty level. Please see Supplement 1 for details on assignment of poverty status in the simulation.

Increasing Investments in Tobacco Control (Sensitivity Analysis)

Background

Comprehensive tobacco control (CTC) programs have been recommended by the Task Force on Community Preventive Services based on evidence that they reduce tobacco use and prevent tobacco-related disease and death.[35] Tobacco control programs combine multiple strategies in a coordinated effort among multiple partners. The strategies employed may include large- and small-scale media, school-based education, quitlines with access to free smoking cessation medications, decreasing tobacco accessibility to minors, and facilitating local adoption of regulations such as clean air policies. While many of these strategies have been evaluated individually, the synergies between strategies are unclear, making it difficult to determine which combination of strategies provides the most cost-effective tobacco control program.

Expenditure elasticity literature

The Community Guide's evidence review organizes the broad evidence base for CTC programs.[36] Most studies provide evaluations of individual state tobacco control programs, particularly in California, Massachusetts and New York (including New York City's program). These studies compare state results pre- and post-implementation or compare outcomes trends in the state to those in other states. Other studies compare tobacco control efforts across all states. These studies use tobacco control expenditures or appropriations as a proxy for intensity of tobacco control and assess the association between expenditures and smoking behaviors.

This latter group of studies is of particular interest in assessing the potential impact of increasing investments in tobacco control. The reported effect sizes allow extrapolation to investments of varying size. Therefore, the simulation model can use the estimated relationship between expense and tobacco use to estimate the effect of each year's investment compared to the base year level. In addition, this literature controls for state tobacco prices.

We focus on cross-state studies that provide estimates of expenditure elasticities we can apply in the simulation model in a manner similar to the price elasticities used to simulate the impact of tobacco taxes. Expenditure elasticities estimate the percent change in smoking behavior with each percent change in tobacco control expenditures. In the literature, expenditure elasticities are generally estimated on cumulative expenditures over current and prior years, because prior years' expenditures may still affect current prevalence. However, studies assume that the impact of prior years' expenditures are less with each successive year and authors have discounted prior years' expenditures by 10-50% per year when tabulating cumulative expenditures.

A series of studies by Farrelly et al. use a consistent methodology to estimate the impact of tobacco control expenditures on youth,[37] young adult[38] and all adult[39] smoking status as summarized in Table S2.3 For all adults, Farrelly et al. discount prior year's expenditures by 10%, 25%, or 50%. We used the estimates with 25% discounting because those results were reported for all age groups. As with price elasticities for tobacco taxes, we simulate the impact on young adults (ages 18-24) through reduced net initiation.

Table S2.3. Expenditure elasticities with respect to smoking status used in ModelHealth:Tobacco

Age group	Estimate	Use in model
**9-17**	-0.059	Change in initiation probability
**18-24**	-0.040	Change in initiation probability
**25+**	-0.016	Change in cessation probability

Calculation of cumulative expenditures

Published studies estimate elasticity based on cumulative expenditures over current and prior years, with the assumption that prior years' expenditures may still affect current year prevalence. However, studies assume that the impact of prior years' expenditures are less each successive year. For

consistency with the measures of effect size used in the simulation model, we use a 25% annual discount of the impact of prior years' expenditures to calculate cumulative expenditures. We measure cumulative expenditures in Year *t* as the sum of $Exp_t + Exp_{t-1} \times (1-.25) + Exp_{t-2} \times (1-.25)^{2}$ +.... through year = t-22, matching the length of the historical series presented in the table below. Note that, due to availability of historical estimates for more years, we use appropriations as a proxy for expenditures. The calculation is illustrated in Table S2.4 for Arkansas in both the status quo, no policy change, scenario and the scenario with expenditures increased to CDC-recommended levels. Note that in the no policy change scenario, per capita expenditures are held at 2021 levels for future years. In the policy change scenario, discounted cumulative expenditures slowly rise as historical expenditures have decreasing influence.

Table S2.4. Cumulative expenditure calculation example, Arkansas					
		No policy change scenario		Increase expenditures to CDC recommended level	
**Model year**	**Calendar year**	**Undiscounted annual per capita appropriations ($2021)**	**Cumulative per capita appropriations, discounted 25% annually**	**Undiscounted annual per capita appropriations ($2021)**	**Cumulative per capita appropriations, discounted 25% annually**
-20	2001	9.70		9.70	
-19	2002	5.07		5.07	
-18	2003	9.63		9.63	
-17	2004	9.67		9.67	
-16	2005	9.22		9.22	
-15	2006	8.92		8.92	
-14	2007	7.52		7.52	
-13	2008	7.31		7.31	
-12	2009	7.26		7.26	
-11	2010	8.30		8.30	
-10	2011	5.65		5.65	
-9	2012	7.92		7.92	
-8	2013	7.65		7.65	
-7	2014	7.45		7.45	
-6	2015	7.09		7.09	
-5	2016	7.17		7.17	
-4	2017	3.91		3.91	
-3	2018	3.77		3.77	
-2	2019	4.78		4.78	
-1	2020	4.40		4.40	
0 (model baseline)	2021	4.14	19.87	4.14	19.87
1	2022	4.14	19.03	14.28	29.16
2	2023	4.14	18.40	14.28	36.14
3	2024	4.14	17.92	14.28	41.36
4	2025	4.14	17.57	14.28	45.28
5	2026	4.14	17.30	14.28	48.22
6	2027	4.14	17.10	14.28	50.42
7	2028	4.14	16.95	14.28	52.08
8	2029	4.14	16.84	14.28	53.32
9	2030	4.14	16.76	14.28	54.25
10	2031	4.14	16.69	14.28	54.95
11	2032	4.14	16.65	14.28	55.48
12	2033	4.14	16.61	14.28	55.87
13	2034	4.14	16.59	14.28	56.16
14	2035	4.14	16.57	14.28	56.39
15	2036	4.14	16.55	14.28	56.55
16	2037	4.14	16.54	14.28	56.68
17	2038	4.14	16.54	14.28	56.78
18	2039	4.14	16.54	14.28	56.86
19	2040	4.14	16.54	14.28	56.91
20	2041	4.14	16.54	14.28	56.95

Applying elasticities to changes in smoking status in the simulation

The relationship between elasticities and relative risk

Relative risk can be computed from elasticities and the change in the policy variables as shown below. We use price elasticities here, but the approach is identical for expenditure elasticities. In all derivations below, we omit subscript *i* to represent individual probabilities of smoking behavior change that vary with demographic characteristics in the microsimulation model. When implemented in the model, the relative impact of a tax and expenditure increase varies by the age groups discussed above but does not vary by other individual characteristics. In upcoming work, we plan to assess variations in policy impact by poverty status.

Price Elasticity (E) (specifically, arc elasticity) can be expressed as:

$$E = \frac{Q' - Q^o}{Q' + Q^o} \Big/ \frac{P' - P^o}{P' + P^o} \qquad \text{[Equation S2.1]}$$

where the Quantity (Q) (smoking prevalence in this application) and Price (P) have superscripts indicated in their initial value (o) and value with policy implementation (‘). Note that in calculating arc elasticities, the denominator in each percentage change is the average of the old and new levels (i.e. (Q' – Q^o) / [(Q'+ Q^o)]/2 and (P' – P^o) / [(P'+P^o)/2] where the 2’s in the percentage change for quantity and price factor-out in the equation for elasticity).

Solving the elasticity formula for Q'yields,

$$Q' = E\left(\frac{P_t - P_o}{P_t + P_o}\right)(Q' + Q^o) + Q^o. \qquad \text{[Equation S2.2]}$$

Dividing both sides by Q^o and rearranging yields,

$$\frac{Q'}{Q^o} = \left\{\frac{1 + E\left(\frac{P' - P^o}{P' + P^o}\right)}{1 - E\left(\frac{P' - P^o}{P' + P^o}\right)}\right\} \qquad \text{[Equation S2.3]}$$

which is the relative risk of smoking with policy change compared to status quo (noting that Q is smoking prevalence).

Policy initiation rates approximated using elasticity

We derived relative risk above to calculate approximate initiation rates for youth and young adult single year of age, sex and race-ethnicity strata inside the simulation as follows:

$$I' \sim I^o \left\{ \frac{1 + E\left(\frac{P' - P^o}{P' + P^o}\right)}{1 - E\left(\frac{P' - P^o}{P' + P^o}\right)} \right\}. \qquad \text{[Equation S2.4]}$$

That is, the policy initiation rate is equal to the original initiation rate times the relative risk.

This is the same approximation applied in prior versions of the SimSmoke model.[40] The elasticities were estimated in the original studies as prevalence elasticities, not as initiation elasticities, and therefore the relative risk reflects prevalence and not initiation relative risks. However, for youth in simulation model, changes in prevalence in the model are the direct result of changes in initiation. Using a difference-in-difference equation analogous to that for cessation below, *I′* can be computed from baseline initiation rates, elasticity, and prior year's smoking prevalence. This computation would require the simulation to calculate and store the macro-variable of prevalence by age, sex and race-ethnicity in each cycle, increasing computation time. In exploratory analyses we found that given low annual initiation rates (they average 0.6% over ages 13-24 in Tobacco Nation states), using Equation S2.4 to reduce computation time only slightly overstates *I′* (and thus slightly understates the decline in youth and young adult smoking prevalence with policy change).

Policy cessation rates approximated using elasticity

Unlike youth, prevalence rates in adults reflect a long process of initiation in younger years and widely varied patterns of cessation and relapse. Therefore, to define the relationship between elasticity and cessation after policy change, we specify a difference-in-difference equation of adult smoking prevalence (difference in time and difference between status quo and policy change scenarios), determine the relationship between cessation rate with policy and elasticity from that equation, and introduce and adjustment to avoid double-counting relapse in the simulation.

We start with the difference-in-difference specification:

$$(Q'_t - Q'_{t-1}) - (Q^o_t - Q^0_{t-1}) = Q^0_{t-1}(1 + C^o)(C' - C^o). \qquad \text{[Equation S2.5]}$$

where Q is smoking prevalence and superscripts are used as above, subscripts indicate the current (t) or past year (t-1), and C is cessation measured as a negative number (i.e. a negative increment to prevalence). The equation states that the difference between the policy scenario and the status quo

scenario in the change of prevalence is equal to the prevalence that would have been observed in time t without policy change [$Q^o_{t-1}(1-C^o)$] multiplied by the marginal effect of the policy-change cessation rate ($C'-C^o$).

With prevalence equal before policy change ($Q'_{t-1} = Q^o_{t-1}$), and solving for C', we obtain

$$C' = C^o + \left(\frac{Q'_t}{Q^0_{t-1}} - \frac{Q^o_t}{Q^0_{t-1}}\right)/(1 - C^o). \qquad \text{[Equation S2.6]}$$

As with computation of the exact expression for initiation in the policy scenario, the simulation model would need to compute and store prevalence rates for age, sex, and race/ethnicity strata each cycle. To increase computation time, we approximate based on small changes in prevalence over time, as Q^o_{t-1} approaches Q^o_t as the change in prevalence approaches zero. Substituting Q^o_t for Q^o_{t-1} yields the approximation

$$C' = C^o + (RR - 1)/(1 - C^o) \qquad \text{[Equation S2.7]}$$

where the relative risk can be calculated using prevalence elasticity as above. The approximation yields a slightly larger cessation rate (in absolute value) for the policy scenario assessed in Tobacco Nation states in this project.

Adjustment to avoid double-counting long-term relapse in the simulation

Equation S2.7 can be read as the status quo cessation rate (C^o) plus the change in cessation attributable to policy change. Elasticities obtained from the literature that are used to calculate the relative risk of smoking with policy change are measured in cross-sectional data comparing smoking status to cigarette prices in the same year that smoking status was reported. Therefore, the elasticity estimates for adults reflect a mixed duration of relapse risk. For the portion of policy-induced quits that occurred in the year smoking status was reported, only short-term relapse is reflected in the elasticity. For these quits, the simulation model's long-term relapse probabilities will be applied appropriately. For the portion of quits that occurred in earlier years, some long-term relapse already occurred. To prevent double-counting of relapse with the model's relapse rates, we adjust the cessation rates attributable to the policy change upward to approximate a short-term cessation rate prior to long-term relapse.

We do not know what portion of long-term relapse from a tax increase occurred by the time smoking status was obtained for measuring elasticity. Conceptually, the net cessation rate that is reflected in the elasticity (C_E) is determined by a higher short-term cessation (C_S) and relapse that has occurred up to the point when the elasticity was estimated (R_E):

$$C_E = C_S - C_S R_E \qquad \text{[Equation S2.8]}$$

which, solved for the short-term cessation rate is:

$$C_S = \frac{C_E}{(1 - R_E)}. \qquad \text{[Equation S2.9]}$$

The important unknown is the relapse percent in the adjustment factor 1/(1- R_E).

We used relapse to the end of the first full year following cessation according to the model's relapse function (R_E = 0.203). The probability of annual cessation in the simulation model was determined by self-reported quits that occurred and were sustained from 6 months to 1 year prior to survey participation. Therefore, we exclude approximately 1 ¾ years of relapse to obtain a policy cessation rate that, when used in the simulation, reduces the extent of double counting of relapse. Although imprecise, we feel this adjustment is better than either 1) failing to adjust for the double-counting of relapse that would occur when the model's relapse rate is applied or 2) making an adjustment that assumes 100% of long-term relapse attributable to a policy change (= 0.671 over ten years in the model's relapse function) has already occurred at the time smoking status was assessed for the estimation of elasticity.

It is intuitive that this adjustment factor be applied to second term of Equation S2.7, but it can also be shown algebraically that the expression for adjusted cessation rate with policy CA':

$$CA' = C^o + \frac{1}{(1 - RE)}(C' - C^o) \quad \text{[Equation S2.10]}$$

(= status quo cessation plus the adjusted margin attributable to policy) solves to

$$CA' = C^o + \frac{1}{(1 - RE)}[(RR - 1)/(1 - C^o)] \qquad \text{[Equation S2.11]}$$

when the expression for C' from Equation S2.7 is substituted in Equation S2.10.

Equation S2.11 is implemented in the simulation model to calculate relapse-adjusted cessation rate with policy change.

References

1. IARC Working Group on the Effectiveness of Tax and Price Policies for Tobacco Control. *IARC Handbooks of Cancer Prevention, Tobacco Control, Vol. 14: Effectiveness of Tax and Price Policies for Tobacco Control.* Lyon, France, 2011.
2. Guide to Community Preventive Services. Tobacco Use and Secondhand Smoke Exposure: Interventions to Increase the Unit Price for Tobacco Products. Centers for Disease Control and Prevention. https://www.thecommunityguide.org/findings/tobacco-use-and-secondhand-smoke-exposure-interventions-increase-unit-price-tobacco-products#GuideReview. Accessed May 18, 2016.
3. Levy DT, Cummings KM, Hyland A. Increasing taxes as a strategy to reduce cigarette use and deaths: results of a simulation model. *Prev Med.* 2000;31(3):279-286.
4. van Baal PH, Brouwer WB, Hoogenveen RT, Feenstra TL. Increasing tobacco taxes: a cheap tool to increase public health. *Health Policy.* 2007;82(2):142-152.
5. Congressional Budget Office. Raising the Excise Tax on Cigarettes: Effects on Health and the Federal Budget. In. Washington: Congressional Budget Office; 2012.
6. Lewit EM, Coate D, Grossman M. The effects of government regulation on teenage smoking. *Journal of Law and Economics.* 1981;24:545-569.
7. Lewit EM, Hyland A, Kerrebrock N, Cummings KM. Price, public policy, and smoking in young people. *Tob Control.* 1997;6 Suppl 2:S17-24.
8. Chaloupka FJ, Pacula RL. Sex and race differences in young people's responsiveness to price and tobacco control policies. *Tob Control.* 1999;8(4):373-377.
9. Harris JE, Chan SW. The continuum-of-addiction: cigarette smoking in relation to price among Americans aged 15-29. *Health Econ.* 1999;8(1):81-86.
10. Tauras JAC, F.J. Price, Clean Indoor Air, and Cigarette Smoking: Evidence from the Longitudinal Data for Young Adults. *NBER Working Papers.* 1999;6937.
11. Emery S, White MM, Pierce JP. Does cigarette price influence adolescent experimentation? *J Health Econ.* 2001;20(2):261-270.
12. Gruber JZ, J. Youth Smoking in the United States: Evidence and Implications. In: Gruber J, ed. *Risky Behavior among Youths.* Chicago: University of Chicago Press; 2001:69-120.
13. Ross H, F.J. C. The effect of public policies and prices on youth smoking. *Southern Economic Journal* 2004;70(4):796-815.
14. DeCicca P, Kenkel D, Mathios A. The fires are not out yet: higher taxes and young adult smoking. *Adv Health Econ Health Serv Res.* 2005;16:293-312.
15. Decicca P, Kenkel D, Mathios A, Shin YJ, Lim JY. Youth smoking, cigarette prices, and anti-smoking sentiment. *Health Econ.* 2008;17(6):733-749.
16. Tauras JA, Markowitz S, Cawley J. Tobacco control policies and youth smoking: evidence from a new era. *Adv Health Econ Health Serv Res.* 2005;16:277-291.
17. Carpenter C, Cook PJ. Cigarette taxes and youth smoking: new evidence from national, state, and local Youth Risk Behavior Surveys. *J Health Econ.* 2008;27(2):287-299.
18. Farrelly MC, Bray JW, Pechacek T, Woollery T. Response by adults to increases in cigarette prices by sociodemographic characteristics. *Southern Economic Journal.* 2001;68(1):156-165.
19. DeCicca P, Kenkel D, Mathios A. Cigarette taxes and the transition from youth to adult smoking: smoking initiation, cessation, and participation. *J Health Econ.* 2008;27(4):904-917.

20. Franz GA. Price effects on the smoking behaviour of adult age groups. *Public Health.* 2008;122(12):1343-1348.
21. Tauras JA. Smoke-free air laws, cigarette prices, and adult cigarette demand. *Economic Inquiry.* 2006;44(2):333–342
22. DeCicca P, McLeod L. Cigarette taxes and older adult smoking: evidence from recent large tax increases. *J Health Econ.* 2008;27(4):918-929.
23. Ong MK, Zhou Q, Sung HY. Sensitivity to cigarette prices among individuals with alcohol, drug, or mental disorders. *Am J Public Health.* 2010;100(7):1243-1245.
24. Smith CE, Hill SE, Amos A. Impact of population tobacco control interventions on socioeconomic inequalities in smoking: a systematic review and appraisal of future research directions. *Tob Control.* 2020;30(e2):e87-95.
25. Brown T, Platt S, Amos A. Equity impact of population-level interventions and policies to reduce smoking in adults: a systematic review. *Drug Alcohol Depend.* 2014;138:7-16.
26. Azagba S, Sharaf M. Cigarette taxes and smoking participation: evidence from recent tax increases in Canada. *Int J Environ Res Public Health.* 2011;8(5):1583-1600.
27. Centers for Disease Control and Prevention. Response to increases in cigarette prices by race/ethnicity, income, and age groups--United States, 1976-1993. *MMWR Morb Mortal Wkly Rep.* 1998;47(29):605-609.
28. Colman GJRDK. Vertical Equity Consequences of Very High Cigarette Tax Increases: If the Poor Are the Ones Smoking, How Could Cigarette Tax Increases be Progressive? *Journal of Policy Analysis and Management.* 2008;27(2):376–400.
29. DeCicca P, McLeod L. Cigarette taxes and older adult smoking: evidence from recent large tax increases. *J Health Econ.* 2008;27(4):918-929.
30. Franks P, Jerant AF, Leigh JP, et al. Cigarette prices, smoking, and the poor: implications of recent trends. *Am J Public Health.* 2007;97(10):1873-1877.
31. Gruber J, Sen A, Stabile M. Estimating price elasticities when there is smuggling: the sensitivity of smoking to price in Canada. *J Health Econ.* 2003;22(5):821-842.
32. Goldin J, Homonoff T. Smoke Gets in Your Eyes: Cigarette Tax Salience and Regressivity. *American Economic Journal: Economic Policy.* 2013;5(1):302-336.
33. Siahpush M, Wakefield MA, Spittal MJ, Durkin SJ, Scollo MM. Taxation reduces social disparities in adult smoking prevalence. *Am J Prev Med.* 2009;36(4):285-291.
34. Farrelly MC, Engelen M. Cigarette prices, smoking, and the poor, revisited. *Am J Public Health.* 2008;98(4):582-583; author reply 583-584.
35. Guide to Community Preventive Services. Tobacco Use and Secondhand Smoke Exposure: Comprehensive Tobacco Control Programs. Centers for Disease Control and Prevention. https://www.thecommunityguide.org/findings/tobacco-use-and-secondhand-smoke-exposure-comprehensive-tobacco-control-programs. Published 2014. Accessed May 18, 2016.
36. Guide to Community Preventive Services. Reducing Tobacco Use and Secondhand Smoke Exposure: Comprehensive Tobacco Control Programs Summary Evidence Table. In: SET-comprehensive.pdf, ed: Centers for Disease Control and Prevention; 2014.
37. Farrelly MC, Loomis BR, Han B, et al. A comprehensive examination of the influence of state tobacco control programs and policies on youth smoking. *Am J Public Health.* 2013;103(3):549-555.
38. Farrelly MC, Loomis BR, Kuiper N, et al. Are tobacco control policies effective in reducing young adult smoking? *J Adolesc Health.* 2014;54(4):481-486.
39. Farrelly MC, Pechacek TF, Thomas KY, Nelson D. The impact of tobacco control programs on adult smoking. *Am J Public Health.* 2008;98(2):304-309.

40. Levy DT, Graham AL, Mabry PL, Abrams DB, Orleans CT. Modeling the impact of smoking-cessation treatment policies on quit rates. *Am J Prev Med.* 2010;38(3 Suppl):S364-372.

SUPPLEMENTAL TABLES

Table A. 20-year effect of $1.50 price increase, compared with no policy change, among NH White and Hispanic individuals, per million persons in 2021, age-adjusted [a]; means of simulations for 30 random number seeds

Population	Change in smoking prevalence at 20 years	Change in SA cancers	Change in SA CVD and diabetes hospitalizations	Change in SA resp. disease hospitalizations	Change in SA cancer deaths	Change in SA CVD and diabetes deaths	Change in SA resp. disease deaths.	Change in SA deaths
Tobacco Nation average[b]								
Non-Hispanic White	-0.43%	-185	-798	-562	-109	-97	-109	-315
Hispanic	-0.38%	-87	-457	-240	-36	-57	-30	-123
Ratio of 20-year cumulative effect: NH White vs Hispanic	0.89	0.47	0.57	0.43	0.33	0.59	0.28	0.39
Non-Tobacco Nation average[b]								
Non-Hispanic White	-0.23%	-139	-422	-283	-76	-39	-61	-176
Hispanic	-0.14%	-54	-213	-150	-28	-23	-18	-69
Ratio of 20-year cumulative effect: NH White vs Hispanic	0.62	0.39	0.50	0.53	0.36	0.59	0.30	0.39

[a] Results reflect the initial simulated model population of 1,000,000 in 2021. The population size changes each year in the model. Population groups are age-adjusted to match the overall age distribution of each state.

[b] The Tobacco Nation average is the average of each state weighted by state adult population. The Non-Tobacco Nation average is computed from the United States average, the Tobacco Nation average and the total population of Tobacco Nation as proportion of the US population.

Table B. Sensitivity analyses of 20-year cumulative effect per million persons of tax increase scenario, compared to static policy scenario, for Tobacco Nation average[b], by poverty status, per million persons in 2021, age-adjusted[a]

Scenario and Population	Change in smoking prevalence at 20 years	Change in SA cancers	Change in SA CVD and diabetes hospitalizations	Change in SA resp. disease hospitalizations	Change in SA cancer deaths	Change in SA CVD and diabetes deaths	Change in SA resp. disease deaths.	Change in SA deaths
Base case								
Below 138% poverty	-0.87%	-498	-2293	-1554	-293	-266	-281	-841
Above 138% poverty	-0.35%	-114	-528	-357	-67	-62	-64	-194
Ratio of 20-year cumulative effect: Below vs above 138% poverty	2.45	4.37	4.34	4.35	4.37	4.29	4.39	4.34
25% lower baseline cessation rates								
Below 138% poverty	-0.93%	-528	-2398	-1639	-306	-285	-287	-878
Above 138% poverty	-0.39%	-123	-530	-371	-72	-64	-65	-202
Ratio of cumulative over 20 years: Below vs above 138% poverty	2.39	4.28	4.52	4.42	4.22	4.43	4.40	4.34
25% higher baseline cessation rates								
Below 138% poverty	-0.80%	-497	-2197	-1513	-288	-250	-267	-805
Above 138% poverty	-0.32%	-105	-468	-329	-65	-61	-60	-186
Ratio of 20-year cumulative effect: Below vs above 138% poverty	2.47	4.71	4.69	4.60	4.46	4.07	4.45	4.33
25% lower relapse rate								
Below 138% poverty	-0.97%	-600	-2648	-1808	-351	-317	-332	-1000
Above 138% poverty	-0.35%	-126	-572	-407	-76	-74	-69	-220
Ratio of cumulative over 20 years: Below vs above 138% poverty	2.74	4.76	4.63	4.44	4.60	4.27	4.80	4.55
25% higher relapse rate								
Below 138% poverty	-0.77%	-442	-2021	-1376	-255	-233	-238	-726
Above 138% poverty	-0.35%	-93	-449	-311	-56	-56	-52	-164
Ratio of 20-year cumulative effect: Below vs above 138% poverty	2.18	4.77	4.50	4.42	4.53	4.16	4.61	4.43
25% lower smoking attributable disease probabilities								
Below 138% poverty	-0.87%	-418	-1877	-1265	-251	-240	-235	-726
Above 138% poverty	-0.35%	-91	-412	-281	-54	-59	-49	-162
Ratio of cumulative over 20 years: Below vs above 138% poverty	2.47	4.58	4.55	4.50	4.64	4.09	4.79	4.49
25% higher smoking attributable disease probability								
Below 138% poverty	-0.86%	-576	-2714	-1842	-332	-286	-318	-937
Above 138% poverty	-0.36%	-133	-612	-418	-77	-73	-65	-215
Ratio of 20-year cumulative effect: Below vs above 138% poverty	2.43	4.34	4.44	4.40	4.32	3.91	4.90	4.35
50% lower price elasticity								
Below 138% poverty	-0.44%	-260	-1161	-813	-153	-139	-147	-439
Above 138% poverty	-0.18%	-54	-252	-186	-30	-35	-30	-96
Ratio of 20-year cumulative effect: Below vs above 138% poverty	2.44	4.82	4.60	4.38	5.05	3.96	4.82	4.58
50% higher price elasticity								
Below 138% poverty	-1.28%	-769	-3397	-2314	-443	-390	-421	-1255
Above 138% poverty	-0.52%	-166	-746	-527	-96	-91	-94	-281

Ratio of 20-year cumulative effect: Below vs above 138% poverty	2.45	4.64	4.55	4.39	4.61	4.28	4.50	4.46
Price elasticity the same for those above and below poverty threshold[c]								
Below 138% poverty	-0.52%	-285	-1278	-882	-166	-154	-160	-479
Above 138% poverty	-0.41%	-145	-663	-471	-84	-85	-84	-253
Ratio of 20-year cumulative effect: Below vs above 138% poverty	1.26	1.97	1.93	1.87	1.98	1.80	1.90	1.89
50% less difference in price elasticity between those above and below poverty threshold[c]								
Below 138% poverty	-0.73%	-426	-1923	-1316	-247	-222	-232	-701
Above 138% poverty	-0.37%	-113	-551	-389	-66	-71	-66	-203
Ratio of 20-year cumulative effect: Below vs above 138% poverty	1.97	3.75	3.49	3.38	3.76	3.11	3.52	3.46
50% greater difference in price elasticity between those above and below poverty threshold[c]								
Below 138% poverty	-1.00%	-609	-2687	-1829	-350	-308	-325	-984
Above 138% poverty	-0.33%	-93	-412	-295	-56	-54	-48	-158
Ratio of 20-year cumulative effect: Below vs above 138% poverty	2.99	6.58	6.53	6.20	6.31	5.69	6.77	6.24

[a] Results reflect the initial simulated model population of 1,000,000 in 2021. The population size changes each year in the model. Population groups are age-adjusted to match the overall age distribution of each state.
[b] The Tobacco Nation average is the average of each state weighted by state adult population.
[c] In scenarios where the differences in elasticities in modified, the differences are specified such that the population weighted mean elasticity remains the same as in the base case. Thus, the elasticity is changed for both population groups.

Table C. Sensitivity analyses of 20-year cumulative effect per million persons of simultaneous tax and tobacco control expenditure increase, compared to static policy scenario, for Tobacco Nation average[b], by poverty status, per million persons in 2021, age-adjusted[a]

Scenario and Population	Change in smoking prevalence at 20 years	Change in SA cancers	Change in SA CVD and diabetes hospitalizations	Change in SA resp. disease hospitalizations	Change in SA cancer deaths	Change in SA CVD and diabetes deaths	Change in SA resp. disease deaths.	Change in SA deaths
Base case								
Below 138% poverty	-7.82%	-3142	-14059	-9750	-1836	-1781	-1875	-5492
Above 138% poverty	-2.84%	-1492	-6499	-4542	-878	-857	-904	-2639
Ratio of 20-year cumulative effect: Below vs above 138% poverty	2.76	2.11	2.16	2.15	2.09	2.08	2.07	2.08
25% lower baseline cessation rates								
Below 138% poverty	-8.71%	-3511	-15412	-10766	-2023	-1944	-2069	-6035
Above 138% poverty	-3.25%	-1622	-7152	-4950	-968	-949	-989	-2905
Ratio of 20-year cumulative effect: Below vs above 138% poverty	2.68	2.16	2.15	2.17	2.09	2.05	2.09	2.08
25% higher baseline cessation rates								
Below 138% poverty	-7.08%	-2917	-12978	-9066	-1689	-1636	-1741	-5066
Above 138% poverty	-2.50%	-1372	-5896	-4167	-820	-807	-851	-2478
Ratio of cumulative over 20 years: Below vs above 138% poverty	2.84	2.13	2.20	2.18	2.06	2.03	2.05	2.04
25% lower relapse rate								
Below 138% poverty	-8.38%	-3438	-15257	-10657	-1983	-1941	-2062	-5986
Above 138% poverty	-2.90%	-1576	-6841	-4802	-936	-912	-977	-2825
Ratio of cumulative over 20 years: Below vs above 138% poverty	2.89	2.18	2.23	2.22	2.12	2.13	2.11	2.12
25% higher relapse rate								
Below 138% poverty	-7.27%	-2920	-12971	-9073	-1678	-1645	-1726	-5049
Above 138% poverty	-2.74%	-1380	-6079	-4278	-816	-815	-858	-2489
Ratio of 20-year cumulative effect: Below vs above 138% poverty	2.65	2.12	2.13	2.12	2.06	2.02	2.01	2.03
25% lower smoking attributable disease probabilities								
Below 138% poverty	-7.92%	-2636	-11257	-7991	-1566	-1548	-1606	-4720
Above 138% poverty	-2.88%	-1226	-5217	-3674	-731	-760	-785	-2276
Ratio of 20-year cumulative effect: Below vs above 138% poverty	2.75	2.15	2.16	2.18	2.14	2.04	2.05	2.07
25% higher smoking attributable disease probability								
Below 138% poverty	-7.75%	-3627	-16642	-11424	-2061	-2002	-2087	-6150
Above 138% poverty	-2.79%	-1723	-7615	-5303	-992	-948	-995	-2935
Ratio of 20-year cumulative effect: Below vs above 138% poverty	2.78	2.10	2.19	2.15	2.08	2.11	2.10	2.10
50% lower price elasticity								
Below 138% poverty	-7.36%	-2809	-12418	-8739	-1607	-1613	-1695	-4915
Above 138% poverty	-2.68%	-1399	-6123	-4277	-826	-834	-871	-2531
Ratio of cumulative over 20 years: Below vs above 138% poverty	2.75	2.01	2.03	2.04	1.95	1.93	1.95	1.94
50% higher price elasticity								
Below 138% poverty	-8.29%	-3522	-15650	-10950	-2029	-1974	-2099	-6101
Above 138% poverty	-2.98%	-1561	-6808	-4761	-927	-909	-957	-2794

Ratio of 20-year cumulative effect: Below vs above 138% poverty	2.78	2.26	2.30	2.30	2.19	2.17	2.19	2.18
50% lower expenditure elasticity								
Below 138% poverty	-4.70%	-1971	-8641	-6025	-1130	-1069	-1148	-3347
Above 138% poverty	-1.70%	-851	-3696	-2599	-501	-496	-531	-1528
Ratio of cumulative over 20 years: Below vs above 138% poverty	2.77	2.32	2.34	2.32	2.26	2.16	2.16	2.19
50% higher expenditure elasticity								
Below 138% poverty	-10.43%	-4227	-18751	-13101	-2446	-2394	-2525	-7366
Above 138% poverty	-3.80%	-2052	-8862	-6230	-1214	-1207	-1256	-3677
Ratio of 20-year cumulative effect: Below vs above 138% poverty	2.74	2.06	2.12	2.10	2.01	1.98	2.01	2.00
Expenditure elasticity 50% higher below poverty threshold than above poverty threshold[c]								
Below 138% poverty	-9.50%	-3868	-17099	-11923	-2232	-2173	-2294	-6699
Above 138% poverty	-2.58%	-1329	-5826	-4063	-786	-786	-814	-2386
Ratio of cumulative over 20 years: Below vs above 138% poverty	3.68	2.91	2.94	2.93	2.84	2.77	2.82	2.81
Price elasticity the same for those above and below poverty threshold[c]								
Below 138% poverty	-7.43%	-2848	-12595	-8860	-1628	-1634	-1715	-4978
Above 138% poverty	-2.88%	-1534	-6704	-4690	-908	-894	-945	-2747
Ratio of 20-year cumulative effect: Below vs above 138% poverty	2.58	1.86	1.88	1.89	1.79	1.83	1.81	1.81
50% less difference in price elasticity between those above and below poverty threshold[c]								
Below 138% poverty	-7.68%	-3057	-13485	-9482	-1756	-1721	-1828	-5305
Above 138% poverty	-2.85%	-1485	-6533	-4571	-881	-876	-922	-2679
Ratio of 20-year cumulative effect: Below vs above 138% poverty	2.70	2.06	2.06	2.07	1.99	1.97	1.98	1.98
50% greater difference in price elasticity between those above and below poverty threshold[c]								
Below 138% poverty	-7.98%	-3306	-14600	-10230	-1897	-1844	-1966	-5708
Above 138% poverty	-2.81%	-1450	-6364	-4445	-859	-856	-901	-2616
Ratio of 20-year cumulative effect: Below vs above 138% poverty	2.84	2.28	2.29	2.30	2.21	2.16	2.18	2.18

[a] Results reflect the initial simulated model population of 1,000,000 in 2021. The population size changes each year in the model. Population groups are age-adjusted to match the overall age distribution of each state.

[b] The Tobacco Nation average is the average of each state weighted by state adult population.

[c] In scenarios where the differences in elasticities in modified, the differences are specified such that the population weighted mean elasticity remains the same as in the base case. Thus, the elasticity is changed for both population groups.

Table D. Sensitivity analyses of 20-year cumulative effect per million persons of tax increase scenario, compared to static policy scenario, for Tobacco Nation average[b], by race, per million persons in 2021, age-adjusted[a]

Scenario and Population	Change in smoking prevalence at 20 years	Change in SA cancers	Change in SA CVD and diabetes hospitalizations	Change in SA resp. disease hospitalizations	Change in SA cancer deaths	Change in SA CVD and diabetes deaths	Change in SA resp. disease deaths.	Change in SA deaths
Base case								
Non-Hispanic Black	-0.31%	-181	-1010	-667	-113	-136	-77	-326
Non-Hispanic White	-0.43%	-185	-798	-562	-109	-97	-109	-315
Ratio of 20-year cumulative effect: NH White vs NH Black	0.71	0.98	1.27	1.19	1.04	1.41	0.70	1.04
25% lower baseline cessation rates								
Non-Hispanic Black	-0.32%	-193	-1060	-694	-117	-138	-82	-336
Non-Hispanic White	-0.48%	-203	-840	-583	-119	-92	-116	-327
Ratio of 20-year cumulative effect: NH White vs NH Black	0.68	0.95	1.26	1.19	0.98	1.49	0.71	1.03
25% higher baseline cessation rates								
Non-Hispanic Black	-0.29%	-169	-963	-639	-103	-126	-78	-307
Non-Hispanic White	-0.39%	-177	-767	-517	-107	-87	-105	-299
Ratio of 20-year cumulative effect: NH White vs NH Black	0.74	0.95	1.26	1.24	0.96	1.45	0.74	1.03
25% lower relapse rate								
Non-Hispanic Black	-0.33%	-203	-1112	-781	-125	-142	-101	-368
Non-Hispanic White	-0.45%	-231	-944	-655	-138	-101	-133	-372
Ratio of 20-year cumulative effect: NH White vs NH Black	0.73	0.88	1.18	1.19	0.91	1.40	0.76	0.99
25% higher relapse rate								
Non-Hispanic Black	-0.28%	-156	-879	-572	-92	-110	-64	-266
Non-Hispanic White	-0.42%	-171	-738	-489	-99	-85	-100	-284
Ratio of 20-year cumulative effect: NH White vs NH Black	0.68	0.91	1.19	1.17	0.92	1.30	0.64	0.94
25% lower smoking attributable disease probabilities								
Non-Hispanic Black	-0.31%	-149	-804	-528	-97	-115	-66	-279
Non-Hispanic White	-0.43%	-154	-672	-450	-94	-92	-94	-279
Ratio of 20-year cumulative effect: NH White vs NH Black	0.71	0.97	1.20	1.17	1.03	1.26	0.71	1.00
25% higher smoking attributable disease probability								
Non-Hispanic Black	-0.30%	-203	-1186	-805	-127	-133	-85	-345
Non-Hispanic White	-0.43%	-218	-984	-672	-128	-106	-135	-369
Ratio of 20-year cumulative effect: NH White vs NH Black	0.70	0.93	1.21	1.20	1.00	1.26	0.63	0.94
50% lower price elasticity								
Non-Hispanic Black	-0.16%	-101	-520	-341	-60	-63	-40	-164
Non-Hispanic White	-0.22%	-97	-430	-297	-54	-42	-61	-157
Ratio of 20-year cumulative effect: NH White vs NH Black	0.70	1.05	1.21	1.15	1.12	1.50	0.66	1.04
50% higher price elasticity								
Non-Hispanic Black	-0.45%	-265	-1501	-1014	-168	-183	-120	-471
Non-Hispanic White	-0.63%	-286	-1219	-815	-165	-127	-169	-461

Ratio of 20-year cumulative effect: NH White vs NH Black	0.71	0.93	1.23	1.24	1.02	1.45	0.71	1.02
Price elasticity the same for those above and below poverty threshold[c]								
Non-Hispanic Black	-0.30%	-178	-983	-665	-109	-129	-80	-319
Non-Hispanic White	-0.43%	-185	-778	-533	-107	-79	-112	-297
Ratio of 20-year cumulative effect: NH White vs NH Black	0.70	0.97	1.26	1.25	1.02	1.64	0.72	1.07
50% less difference in price elasticity between those above and below poverty threshold[c]								
Non-Hispanic Black	-0.30%	-177	-991	-636	-107	-124	-77	-308
Non-Hispanic White	-0.43%	-188	-788	-533	-110	-85	-109	-304
Ratio of 20-year cumulative effect: NH White vs NH Black	0.70	0.94	1.26	1.19	0.98	1.45	0.71	1.01
50% greater difference in price elasticity between those above and below poverty threshold[c]								
Non-Hispanic Black	-0.30%	-178	-1021	-653	-107	-123	-80	-310
Non-Hispanic White	-0.43%	-196	-828	-554	-115	-88	-112	-315
Ratio of 20-year cumulative effect: NH White vs NH Black	0.70	0.91	1.23	1.18	0.93	1.40	0.71	0.98

[a] Results reflect the initial simulated model population of 1,000,000 in 2021. The population size changes each year in the model. Population groups are age-adjusted to match the overall age distribution of each state.

[b] The Tobacco Nation average is the average of each state weighted by state adult population.

[c] In scenarios where the differences in elasticities in modified, the differences are specified such that the population weighted mean elasticity remains the same as in the base case. Thus, the elasticity is changed for both population groups.

Table E. Sensitivity analyses of 20-year cumulative effect per million persons of simultaneous tax and TCE increase, compared to static policy scenario, for Tobacco Nation average[b], by race, per million persons in 2021, age-adjusted[a]

Scenario and Population	Change in smoking prevalence at 20 years	Change in SA cancers	Change in SA CVD and diabetes hospitalizations	Change in SA resp. disease hospitalizations	Change in SA cancer deaths	Change in SA CVD and diabetes deaths	Change in SA resp. disease deaths.	Change in SA deaths
Base case								
Non-Hispanic Black	-3.13%	-1787	-9434	-6355	-1126	-1432	-819	-3377
Non-Hispanic White	-3.82%	-1829	-7395	-5394	-1061	-966	-1158	-3185
Ratio of 20-year cumulative effect: NH White vs NH Black	0.82	0.98	1.28	1.18	1.06	1.48	0.71	1.06
25% lower baseline cessation rates								
Non-Hispanic Black	-3.46%	-1939	-10279	-6983	-1234	-1535	-932	-3701
Non-Hispanic White	-4.38%	-2079	-8151	-5903	-1217	-1048	-1266	-3532
Ratio of 20-year cumulative effect: NH White vs NH Black	0.79	0.93	1.26	1.18	1.01	1.46	0.74	1.05
25% higher baseline cessation rates								
Non-Hispanic Black	-2.84%	-1682	-8827	-5967	-1084	-1315	-755	-3155
Non-Hispanic White	-3.37%	-1711	-6888	-4951	-988	-871	-1047	-2906
Ratio of 20-year cumulative effect: NH White vs NH Black	0.84	0.98	1.28	1.21	1.10	1.51	0.72	1.09
25% lower relapse rate								
Non-Hispanic Black	-3.30%	-1931	-10096	-6935	-1232	-1530	-896	-3658
Non-Hispanic White	-3.95%	-2017	-7895	-5728	-1184	-1013	-1214	-3411
Ratio of 20-year cumulative effect: NH White vs NH Black	0.84	0.96	1.28	1.21	1.04	1.51	0.74	1.07
25% higher relapse rate								
Non-Hispanic Black	-2.92%	-1648	-8797	-5963	-1046	-1334	-753	-3133
Non-Hispanic White	-3.67%	-1780	-7010	-5017	-1034	-895	-1047	-2976
Ratio of 20-year cumulative effect: NH White vs NH Black	0.80	0.93	1.26	1.19	1.01	1.49	0.72	1.05
25% lower smoking attributable disease probabilities								
Non-Hispanic Black	-3.21%	-1479	-7634	-5242	-964	-1239	-721	-2925
Non-Hispanic White	-3.89%	-1523	-5993	-4399	-901	-867	-992	-2760
Ratio of 20-year cumulative effect: NH White vs NH Black	0.83	0.97	1.27	1.19	1.07	1.43	0.73	1.06
25% higher smoking attributable disease probability								
Non-Hispanic Black	-3.04%	-2052	-11107	-7404	-1287	-1575	-886	-3748
Non-Hispanic White	-3.77%	-2169	-8775	-6326	-1209	-1002	-1268	-3479
Ratio of 20-year cumulative effect: NH White vs NH Black	0.81	0.95	1.27	1.17	1.06	1.57	0.70	1.08
50% lower price elasticity								
Non-Hispanic Black	-2.96%	-1664	-8723	-5885	-1069	-1328	-761	-3158
Non-Hispanic White	-3.63%	-1789	-6931	-5008	-1040	-887	-1062	-2988
Ratio of 20-year cumulative effect: NH White vs NH Black	0.82	0.93	1.26	1.18	1.03	1.50	0.72	1.06
50% higher price elasticity								
Non-Hispanic Black	-3.27%	-1927	-10185	-6918	-1237	-1518	-873	-3628
Non-Hispanic White	-4.02%	-2034	-8010	-5785	-1181	-1004	-1225	-3411

Ratio of 20-year cumulative effect: NH White vs NH Black	0.81	0.95	1.27	1.20	1.05	1.51	0.71	1.06
50% lower expenditure elasticity								
Non-Hispanic Black	-4.70%	-1971	-8641	-6025	-1130	-1069	-1148	-3347
Non-Hispanic White	-1.70%	-851	-3696	-2599	-501	-496	-531	-1528
Ratio of 20-year cumulative effect: NH White vs NH Black	2.77	2.32	2.34	2.32	2.26	2.16	2.16	2.19
50% higher expenditure elasticity								
Non-Hispanic Black	-4.19%	-2424	-12912	-8773	-1546	-1942	-1130	-4618
Non-Hispanic White	-5.14%	-2554	-10136	-7394	-1485	-1275	-1587	-4346
Ratio of 20-year cumulative effect: NH White vs NH Black	0.82	0.95	1.27	1.19	1.04	1.52	0.71	1.06
Expenditure elasticity 50% higher below poverty threshold than above poverty threshold[c]								
Non-Hispanic Black	-3.09%	-1761	-9371	-6339	-1122	-1412	-802	-3336
Non-Hispanic White	-3.81%	-1900	-7473	-5432	-1107	-952	-1141	-3200
Ratio of 20-year cumulative effect: NH White vs NH Black	0.81	0.93	1.25	1.17	1.01	1.48	0.70	1.04
Price elasticity the same for those above and below poverty threshold[c]								
Non-Hispanic Black	-3.11%	-1790	-9392	-6383	-1153	-1408	-820	-3382
Non-Hispanic White	-3.81%	-1897	-7407	-5349	-1102	-934	-1132	-3168
Ratio of 20-year cumulative effect: NH White vs NH Black	0.82	0.94	1.27	1.19	1.05	1.51	0.72	1.07
50% less difference in price elasticity between those above and below poverty threshold[c]								
Non-Hispanic Black	-3.11%	-1794	-9422	-6386	-1154	-1408	-817	-3379
Non-Hispanic White	-3.81%	-1903	-7424	-5347	-1111	-943	-1133	-3187
Ratio of 20-year cumulative effect: NH White vs NH Black	0.82	0.94	1.27	1.19	1.04	1.49	0.72	1.06
50% greater difference in price elasticity between those above and below poverty threshold[c]								
Non-Hispanic Black	-3.12%	-1797	-9473	-6398	-1150	-1415	-811	-3376
Non-Hispanic White	-3.82%	-1917	-7482	-5397	-1118	-951	-1139	-3208
Ratio of 20-year cumulative effect: NH White vs NH Black	0.82	0.94	1.27	1.19	1.03	1.49	0.71	1.05

[a] Results reflect the initial simulated model population of 1,000,000 in 2021. The population size changes each year in the model. Population groups are age-adjusted to match the overall age distribution of each state.
[b] The Tobacco Nation average is the average of each state weighted by state adult population.
[c] In scenarios where the differences in elasticities in modified, the differences are specified such that the population weighted mean elasticity remains the same as in the base case. Thus, the elasticity is changed for both population groups.

© 2025 Donovan E.M. et al.
